# Identification of Spiro-Fused Pyrrolo[3,4-*a*]pyrrolizines and Tryptanthrines as Potential Antitumor Agents: Synthesis and In Vitro Evaluation

**DOI:** 10.3390/ijms222111997

**Published:** 2021-11-05

**Authors:** Diana K. Latypova, Stanislav V. Shmakov, Sofya A. Pechkovskaya, Alexander S. Filatov, Alexander V. Stepakov, Nickolay A. Knyazev, Vitali M. Boitsov

**Affiliations:** 1 Saint-Petersburg National Research Academic University of the Russian Academy of Sciences, 194021 Saint-Petersburg, Russia; dialat00@mail.ru (D.K.L.); stas-svs@list.ru (S.V.S.); 2 Institute of Cytology, Russian Academy of Sciences, 194064 Saint-Petersburg, Russia; sapechkovskaya@gmail.com; 3 Department of Chemistry, Saint-Petersburg State University, 199034 Saint Petersburg, Russia; shurikfilatov@yandex.ru (A.S.F.); alstepakov@yandex.ru (A.V.S.); 4 Department of Organic Chemistry, Saint Petersburg State Institute of Technology, 190013 Saint-Petersburg, Russia; 5 Saint-Petersburg Clinical Scientific and Practical Center for Specialized Types of Medical Care (Oncological), 197758 Saint-Petersburg, Russia

**Keywords:** one-pot synthesis, 1,3-dipolar cycloaddition, tryptanthrin-derived azomethine ylide, pyrrolo[3,4-*a*]pyrrolizine, tumor cells, antiproliferative activity, cell motility, morphological changes (cytoskeleton), cell cycle, cell death

## Abstract

A series of heterocyclic compounds containing a spiro-fused pyrrolo[3,4-*a*]pyrrolizine and tryptanthrin framework have been synthesized and studied as potential antitumor agents. Cytotoxicity of products was screened against human erythroleukemia (K562) and human cervical carcinoma (HeLa) cell lines. Among the screened compounds. **4a**, **4b** and **5a** were active against human erythroleukemia (K562) cell line, while **4a** and **5a** were active against cervical carcinoma (HeLa) cell line. In agreement with the DNA cytometry studies, the tested compounds have achieved significant cell-cycle perturbation with higher accumulation of cells in G2/M phase and induced apoptosis. Using confocal microscopy, we found that with **4a** and **5a** treatment of HeLa cells, actin filaments disappeared, and granular actin was distributed diffusely in the cytoplasm in 76–91% of cells. We discovered that HeLa cells after treatment with compounds **4a** and **5a** significantly reduced the number of cells with filopodium-like membrane protrusions (from 63 % in control cells to 29% after treatment) and a decrease in cell motility.

## 1. Introduction

Oncological diseases are one of the most common public health problems and the second leading cause of death after cardiovascular disease. Increased drug resistance and the emergence of tumor resistance as well as severe side-effects of chemotherapeutic agents reduce the clinical efficacy of currently used anticancer drugs and treatments. Moreover, multi-drug resistance of malignant tumors is one of the main causes for their clinical progression. Despite the increasing use of targeted drugs and methods of immunotherapy of oncological diseases, the development of cytostatic agents remains an important challenge for the treatment of cancer. At the same time, the emergence of tumor resistance requires the creation of cytostatics that are not just derivatives of “classical” drugs, but originate from compounds of a new nature. It is also worth noting that the ability of cytostatics to influence the activity of the immune system makes it possible to use them for the treatment of autoimmune diseases.

Natural products or synthetic compounds inspired from natural products continue to be excellent sources for new drug candidates. Many of the most currently applicable (or that under clinical trials) anticancer drugs are either themselves compounds of natural origin, or are designed based on naturally occurring compounds [1,2]. In most cases, these are compounds of complex structure that can be obtained only as a result of complex multistage synthesis. Recent advances in the synthesis of such complex heterocyclic systems have led to a significant increase in interest in the development of efficient methods for the synthesis of various derivatives and structural analogues of these compounds as potential drugs or biological probes [3,4]. Therefore, oxindole, azabicyclohexane, pyrrolizine, pyrroloisoquinoline and indoloquinazoline units are heterocyclic motifs that form the core of a large family of alkaloid natural products with strong bioactivity profiles and interesting structural properties [5]. Compounds comprising pyrrolizine moiety showed antitumor [6,7,8,9], anti-inflammatory [8], antiviral [9], antimicrobial [10,11,12], antibacterial [13], antimycobacterial [14], antitubercular [15], antifungal [16] activity. Pyrrolo[3,4-*a*]pyrrolizine derivatives are known as inhibitors of serine protease from the blood coagulation cascade [17,18,19]. They also may be used for treating CFTR (cystic fibrosis transmembrane conductance regulator) mediated diseases, in particular cystic fibrosis [20]. Various biological activities have been found for indolo[2,1-*b*]quinazolin-6,12-dione (tryptanthrin) and its analogues, e.g., anti-tumor [21], immune-modulatory [22], anti-inflammatory [23], antimicrobial [24], antileishmanial [25], antifungal [26], antimalarial [27], cytotoxic [28] and activity as indoleamine 2,3-dioxygenase (IDO-1) inhibitor [29] and antitubercular agent [30]. It was previously shown that 8-methyltryptanthrin successfully differentiated P19CL6 cells into spontaneously beating cardiomyocyte-like cells [31]. Liao and Leung found that tryptanthrin might cause growth inhibition of the human neuroblastoma LA-N-1 cells [32].

In our recent works it was shown that (spiro) azabicyclo[3.1.0]hexanes and (spiro) cyclopropa[*a*]pyrrolizines are readily available from cyclopropenes and azomethine ylides (generated in situ from amino acids or peptides and carbonyl compounds such as ninhydrins, indenoquinoxalines, pyrroloisoquinolines and tryptanthrines) and some adducts inhibited the cancer cells grows in vitro [33,34,35,36,37]. In addition, the morphological changes of cells occurring under the action of these compounds (in particular, such cytoskeleton components as actin microfilaments) suggest that the studied substances have not only cytostatic activity, but also can lead to a decrease in metastasis of tumor cells, which opens up broad prospects for the study of their action in vivo [38].

We here report that one-pot three-component 1,3-dipolar cycloaddition reactions of various maleimides with in situ-generated tryptanthrin-derived azomethine ylide lead to the formation of two diastereomeric products with pyrrolo[3,4-*a*]pyrrolizine moiety. All the compounds were evaluated for their antiproliferative activity as well as cell motility; morphological changes, cell cycle, and cell death were evaluated for the most active products.

## 2. Materials and Methods

### 2.1. Synthesis

NMR spectra were recorded with a Bruker Avance 400 spectrometer (400.13 MHz for ^1^H and 100.61 MHz for ^13^C). Chemical shifts are reported in ppm relative to residual CHCl_3_ (^1^H, δ = 7.26 ppm), CDCl_3_ (^13^C, δ = 77.17 ppm) and residual DMSO-d_6_ (^1^H, δ = 2.50 ppm), DMSO-d_6_ (^13^C, δ = 39.52 ppm) as internal standards. All ^13^C NMR spectra were proton-decoupled. Mass spectra were recorded with a HRMS-ESIQTOF mass-analyzer, electrospray ionization, positive mode. Single crystal X-ray diffraction experiments for **4c** and **5d** were carried out with a Bruker «ApexDuo» diffractometer using monochromated Mo Kα radiation. All chemicals were purchased from commercial suppliers and used without further purification. Preparative thin layer chromatography (PTLC) was performed using Merck silica gel 60. Commercial grade solvents and reagents were used without further purification. Analytical thin layer chromatography (TLC) was performed using Merck 60 F254 silica gel plates to monitor the reaction progress. Maleimides **2a**-**d** were synthesized according to known procedures [39].

One-Pot Three-Component Reaction of Tryptanthrin, Maleimides and Proline as an α-Amino Acid; General Procedure

A mixture of tryptanthrin **1** (0.8 mmol), corresponding maleimide **2a**-**d** (0.8 mmol), and α-amino acid **3** (1.2 mmol) was suspended in a mixture of methanol–benzene (3:1, 8 mL). After heating to reflux for 24 h (progress was monitored by TLC), the reaction mixture was cooled to room temperature and all volatiles were removed under reduced pressure. The residue was transferred into a separatory funnel using dichloromethane (5 mL), and washed twice with water and brine. The washed organic phase was dried over anhydrous sodium sulfate, filtered and concentrated in vacuo. The resulting crude product was purified by PTLC using a mixture of hexane–dichloromethane (2:1) to afford the pure title cycloadducts **4** and **5**.

(±)-(3a′*S*,4′*R*,8a′*R*,8b′*R*)-6′,7′,8′,8a′-tetrahydro-1′*H*,12*H*-spiro[indolo[2,1-*b*]quinazoline-6,4′-pyrrolo[3,4-*a*]pyrrolizine]-1′,3′,12(2′*H*,3a′*H*,8b′*H*)-trione (**4a**) and (±)-(3a′*S*,4′*S*,8a′*S*,8b′*R*)-6′,7′,8′,8a′-tetrahydro-1′*H*,12*H*-spiro[indolo[2,1-*b*]quinazoline-6,4′-pyrrolo[3,4-*a*]pyrrolizine]-1′,3′,12(2′*H*,3a′*H*,8b′*H*)-trione (**5a**). 1,3-Dipolar cycloadducts **4a** and **5a** were obtained by following General Procedure from tryptanthrin (**1**; 200 mg, 0.8 mmol), maleimide **2a** (78 mg, 0.8 mmol) and *L*-proline (**3**; 139 mg, 1.2 mmol). Yield: 109 mg (34%) **4a** and 95 mg (30%) **5a** as colorless solids. (**4a**): mp >230 °C (dec.), R_f_ = 0.55 (SiO_2_, petr. ether/ethyl acetate, 1:1). ^1^H NMR (400 MHz, CDCl_3_): δ = 8.63 (d, J = 8.0 Hz, 1 H, H_arom_), 8.43 (d, J = 8.0 Hz, 1 H, H_arom_), 8.33 (s, 1 H, NH), 7.84–7.73 (m, 2 H, H_arom_), 7.61–7.50 (m, 2 H, H_arom_), 7.38–7.30 (m, 2 H, H_arom_), 4.72–4.64 (m, 1 H, CH), 4.01 (d, J = 7.7 Hz, 1 H, CH), 3.72 (t, J = 7.7 Hz, 1 H, CH), 2.50–2.42 (m, 1 H, CH_2_), 2.22–2.13 (m, 1 H, CH_2_), 2.13–1.90 (m, 4 H, CH_2_). ^13^C NMR (101 MHz, CDCl_3_): δ = 176.1, 174.5, 159.5, 159.4, 146.8, 139.9, 134.4, 130.7, 128.0, 127.5, 127.0, 126.7, 126.2, 126.1, 121.8, 117.1, 68.9, 64.7, 60.4, 46.1, 42.2, 26.4, 23.2. HRMS-ESI: m/z [M + H]^+^ calcd for C_23_H_19_N_4_O_3_^+^: 399.1457; found: 399.1452. (**5a**): m.p. 247–250 °C (dec.), R_f_ = 0.67 (SiO_2_, petr. ether/ethyl acetate, 1:1). ^1^H NMR (400 MHz, CDCl_3_): δ = 8.63 (d, J = 8.1 Hz, 1 H, H_arom_), 8.44 (d, J = 7.9 Hz, 1 H, H_arom_), 8.13 (s, 1 H, NH), 7.85–7.73 (m, 2 H, H_arom_), 7.60–7.50 (m, 2 H, H_arom_), 7.38–7.30 (m, 2 H, H_arom_), 4.68 (q, J = 7.4 Hz, 1 H, CH), 4.01 (d, J = 7.7 Hz, 1 H, CH), 3.72 (t, J = 7.6 Hz, 1 H, CH), 2.50–2.41 (m, 1 H, CH_2_), 2.22–2.13 (m, 1 H, CH_2_), 2.13–1.90 (m, 4 H, CH_2_). ^13^C NMR (101 MHz, CDCl_3_): δ = 176.4, 174.7, 159.5, 159.4, 146.8, 139.8, 134.4, 130.7, 127.9, 127.5, 127.0, 126.8, 126.3, 126.1, 121.8, 117.1, 68.9, 64.7, 60.3, 46.1, 42.2, 26.4, 23.3. HRMS-ESI: m/z [M + H]^+^ calcd for C_23_H_19_N_4_O_3_^+^: 399.1457; found: 399.1452.

(±)-(3a′*S*,4′*R*,8a′*R*,8b′*R*)-2′-ethyl-6′,7′,8′,8a′-tetrahydro-1′*H*,12*H*-spiro[indolo[2,1-*b*]quinazoline-6,4′-pyrrolo[3,4-*a*]pyrrolizine]-1′,3′,12(2′*H*,3a′*H*,8b′*H*)-trione (**4b**) and (±)-(3a′*S*,4′*S*,8a′*S*,8b′*R*)-2′-ethyl-6′,7′,8′,8a′-tetrahydro-1′*H*,12*H*-spiro[indolo[2,1-*b*]quinazoline-6,4′-pyrrolo[3,4-*a*]pyrrolizine]-1′,3′,12(2′*H*,3a′*H*,8b′*H*)-trione (**5b**). 1,3-Dipolar cycloadducts **4b** and **5b** were obtained by following General Procedure from tryptanthrin (**1**; 200 mg, 0.8 mmol), maleimide **2b** (101 mg, 0.8 mmol) and *L*-proline (**3**; 139 mg, 1.2 mmol). Yield: 100 mg (29%) **4b** and 80 mg (23%) **5b** as colorless solids. (**4b**): mp 211–213 °C, R_f_ = 0.31 (SiO_2_, petr. ether/ethyl acetate, 1:1). ^1^H NMR (400 MHz, DMSO-d_6_): δ = 8.50 (d, J = 7.8 Hz, 1 H, H_arom_), 8.31 (d, J = 8.0 Hz, 1 H, H_arom_), 7.93–7.86 (m, 1 H, H_arom_), 7.82–7.78 (m, 1 H, H_arom_), 7.66–7.60 (m, 1 H, H_arom_), 7.60–7.54 (m, 1 H, H_arom_), 7.40–7.35 (m, 1 H, H_arom_), 7.16–7.12 (m, 1 H, H_arom_), 4.62–4.52 (m, 1 H, CH), 4.05 (d, J = 7.7 Hz, 1 H, CH), 3.66 (t, J = 7.6 Hz, 1 H, CH), 3.51 (q, J = 7.2 Hz, 2 H, CH_2_), 2.32–2.24 (m, 1 H, CH_2_), 2.15–2.07 (m, 1 H, CH_2_), 1.99–1.75 (m, 4 H, CH_2_) 1.18 (t, J = 7.2 Hz, 3 H, CH_3_). ^13^C NMR (101 MHz, DMSO-d_6_): δ = 176.6, 175.1, 160.1, 159.4, 146.9, 139.9, 135.1, 130.6, 128.3, 127.9, 127.1, 126.8, 126.3, 121.9, 116.4, 69.1, 64.7, 59.1, 46.9, 42.4, 33.8, 26.3, 23.5, 13.4. HRMS-ESI: *m*/*z* [M + H]^+^ calcd for C_25_H_23_N_4_O_3_^+^: 427.1771; found: 427.1765. (**5b**): m.p. 165–167 °C, R_f_ = 0.46 (SiO_2_, petr. ether/ethyl acetate, 1:1). ^1^H NMR (400 MHz, DMSO-d_6_): δ = 8.51 (d, J = 8.0 Hz, 1 H, H_arom_), 8.28 (d, J = 7.8 Hz, 1 H, H_arom_), 7.94 (d, J = 7.5 Hz, 1 H, H_arom_), 7.91–7.82 (m, 1 H, H_arom_), 7.72–7.45 (m, 4 H, H_arom_), 4.70–4.58 (m, 1 H, CH), 4.42 (d, J = 9.8 Hz, 1 H, CH), 3.63 (dd, J = 9.7 and 6.9 Hz, 1 H, CH), 3.33–3.17 (m, 2 H, CH_2_), 3.11–2.97 (m, 1 H, CH_2_), 2.58–2.45 (m, 1 H, CH_2_), 2.30–2.15 (m, 1 H, CH_2_), 2.07–1.90 (m, 2 H, CH_2_), 1.75–1.55 (m, 1 H, CH_2_) 0.91 (t, J = 7.1 Hz, 3 H, CH_3_). ^13^C NMR (101 MHz, DMSO-d_6_): δ = 178.1, 175.9, 159.9, 159.0, 146.4, 139.9, 135.5, 131.0, 129.6, 128.2, 128.0, 127.2, 127.0, 121.3, 116.7, 73.4, 65.8, 59.9, 53.8, 49.1, 33.6, 30.2, 25.4, 13.1. HRMS-ESI: *m*/*z* [M + H]^+^ calcd for C_25_H_23_N_4_O_3_^+^: 427.1772; found: 427.1765.

(±)-(3a′*S*,4′*R*,8a′*R*,8b′*R*)-2′-phenyl-6′,7′,8′,8a′-tetrahydro-1′*H*,12*H*-spiro[indolo[2,1-*b*]quinazoline-6,4′-pyrrolo[3,4-*a*]pyrrolizine]-1′,3′,12(2′*H*,3a′*H*,8b′*H*)-trione (**4c**) and (±)-(3a′*S*,4′*S*,8a′*S*,8b′*R*)-2′-phenyl-6′,7′,8′,8a′-tetrahydro-1′*H*,12*H*-spiro[indolo[2,1-*b*]quinazoline-6,4′-pyrrolo[3,4-*a*]pyrrolizine]-1′,3′,12(2′*H*,3a′*H*,8b′*H*)-trione (**5c**). 1,3-Dipolar cycloadducts **4c** and **5c** were obtained by following General Procedure from tryptanthrin (**1**; 200 mg, 0.8 mmol), maleimide **2c** (140 mg, 0.8 mmol) and *L*-proline (**3**; 139 mg, 1.2 mmol). Yield: 28 mg (7%) **4c** and 28 mg (7%) **5c** as colorless solids. (**4c**): R_f_ = 0.42 (SiO_2_, petr. ether/ethyl acetate, 1:1). ^1^H NMR (400 MHz, CDCl_3_): δ = 8.64 (d, J = 8.1 Hz, 1 H, H_arom_), 8.44 (d, J = 7.8 Hz, 1 H, H_arom_), 7.87–7.76 (m, 2 H, H_arom_), 7.64–7.23 (m, 9 H, H_arom_), 4.86–4.75 (m, 1 H, CH), 4.15 (d, J = 7.9 Hz, 1 H, CH), 3.85 (t, J = 7.8 Hz, 1 H, CH), 2.57–2.45 (m, 1 H, CH_2_), 2.34–2.23 (m, 1 H, CH_2_), 2.16–1.92 (m, 4 H, CH_2_). ^13^C NMR (101 MHz, CDCl_3_): δ = 175.5, 173.8, 159.5, 146.8, 140.0, 134.4, 131.9, 130.6, 129.3, 128.8, 128.0, 127.5, 127.0, 126.7, 126.5, 126.3, 126.1, 121.8, 117.2, 69.9, 65.3, 58.8, 45.1, 43.0, 26.3, 23.6. HRMS-ESI: m/z [M + H]^+^ calcd for C_29_H_23_N_4_O_3_^+^: 475.1771; found: 475.1765. (**5c**) R_f_ = 0.52 (SiO_2_, petr. ether/ethyl acetate, 1:1). ^1^H NMR (400 MHz, CDCl_3_): δ = 8.67 (d, J = 7.8 Hz, 1 H, H_arom_), 8.40 (d, J = 7.9 Hz, 1 H, H_arom_), 7.76–7.25 (m, 9 H, H_arom_), 7.20–7.11 (m, 2 H, H_arom_), 5.08–4.95 (m, 1 H, CH), 4.33 (d, J = 10.1 Hz, 1 H, CH), 3.67 (dd, J = 10.1 and 6.7 Hz, 1 H, CH), 3.00–2.90 (m, 1 H, CH_2_), 2.83–2.72 (m, 1 H, CH_2_), 2.54–2.40 (m, 1 H, CH_2_), 2.16–2.03 (m, 2 H, CH_2_), 1.95–1.82 (m, 1 H, CH_2_). ^13^C NMR (101 MHz, CDCl_3_): δ = 176.7, 174.1, 159.5, 146.6, 140.6, 134.3, 131.8, 130.9, 128.9, 128.4, 127.9, 127.5, 127.0, 126.7, 126.4, 126.3, 125.2, 122.1, 117.6, 73.9, 66.3, 55.3, 53.6, 49.2, 30.4, 25.5. HRMS-ESI: m/z [M + H]^+^ calcd for C_29_H_23_N_4_O_3_^+^: 475.1772; found: 475.1765.

(±)-(3a′*S*,4′*R*,8a′*R*,8b′*R*)-2′-phenethyl-6′,7′,8′,8a′-tetrahydro-1′*H*,12*H*-spiro[indolo[2,1-*b*]quinazoline-6,4′-pyrrolo[3,4-*a*]pyrrolizine]-1′,3′,12(2′*H*,3a′*H*,8b′*H*)-trione (**4d**) and (±)-(3a′*S*,4′*S*,8a′*S*,8b′*R*)-2′-phenethyl-6′,7′,8′,8a′-tetrahydro-1′*H*,12*H*-spiro[indolo[2,1-*b*]quinazoline-6,4′-pyrrolo[3,4-*a*]pyrrolizine]-1′,3′,12(2′*H*,3a′*H*,8b′*H*)-trione (**5d**). 1,3-Dipolar cycloadducts **4d** and **5d** were obtained by following General Procedure from tryptanthrin (**1**; 200 mg, 0.8 mmol), maleimide **2d** (162 mg, 0.8 mmol) and *L*-proline (**3**; 139 mg, 1.2 mmol). Yield: 80 mg (20%) **4d** and 45 mg (11%) **5d** as colorless solids. (**4d**): m.p. 198–201 °C, R_f_ = 0.20 (SiO_2_, petr. ether/ethyl acetate, 2:1). ^1^H NMR (400 MHz, CDCl_3_): δ = δ = 8.61 (d, J = 8.0 Hz, 1 H, H_arom_), 8.42 (dd, J = 8.0 and 1.1 Hz, 1 H, H_arom_), 7.85–7.71 (m, 2 H, H_arom_), 7.60–7.45 (m, 2 H, H_arom_), 7.40–7.20 (m, 6 H, H_arom_), 6.75 (d, J = 7.6 Hz, 1 H, H_arom_), 4.74–4.62 (m, 1 H, CH), 4.02–3.80 (m, 3 H, CH + CH_2_), 3.61 (t, J = 7.8 Hz, 1 H, CH), 3.15–2.95 (m, 2 H, CH_2_), 2.37–2.26 (m, 1 H, CH_2_), 2.17–1.82 (m, 5 H, CH_2_). ^13^C NMR (101 MHz, CDCl_3_): δ = 176.5, 174.7, 159.5, 146.8, 139.9, 137.7, 134.4, 130.4, 129.1, 128.6, 127.9, 127.4, 126.94, 126.88, 126.6, 126.5, 125.9, 121.8, 116.9, 69.2, 64.6, 58.5, 44.8, 42.5, 40.2, 33.4, 26.2, 23.5. HRMS-ESI: *m*/*z* [M + H]^+^ calcd for C_31_H_27_N_4_O_3_^+^: 503.2084; found: 503.2078. (**5d**): m.p. 166–168 °C, R_f_ = 0.60 (SiO_2_, petr. ether/ethyl acetate, 2:1). ^1^H NMR (400 MHz, CDCl_3_): δ = 8.67 (d, J = 8.0 Hz, 1 H, H_arom_), 8.39 (d, J = 7.8 Hz, 1 H, H_arom_), 7.75–7.35 (m, 6 H, H_arom_), 7.33–7.10 (m, 5 H, H_arom_), 4.95–4.84 (m, 1 H, CH), 4.15 (d, J = 9.9 Hz, 1 H, CH), 3.73–3.42 (m, 3 H, CH + CH_2_), 2.97–2.82 (m, 2 H, CH_2_), 2.78–2.62 (m, 2 H, CH_2_), 2.49–2.36 (m, 1 H, CH_2_), 2.14–1.80 (m, 3 H, CH_2_). ^13^C NMR (101 MHz, CDCl_3_): δ = 177.5, 174.9, 159.5, 159.0, 146.5, 140.5, 140.0, 134.4, 130.8, 128.7, 128.5, 128.4, 127.6, 127.4, 127.0, 126.6, 126.4, 125.1, 121.9, 117.6, 73.2, 65.9, 55.7, 53.3, 48.8, 40.1, 33.6, 30.3, 25.5. HRMS-ESI: *m*/*z* [M + H]^+^ calcd for C_31_H_27_N_4_O_3_^+^: 503.2084; found: 503.2078.

### 2.2. Cell Culture and Culturing Conditions

Human cervical carcinoma (HeLa) and human erythroleukemia (K-562) cell lines were obtained from the Collection of Cell Cultures of Vertebrates (Institute of Cytology, Russian Academy of Sciences, St. Peterburg). HeLa cells were cultured in DMEM (HyClone, South Logan, UT, USA) supplemented with 10% fetal bovine serum (HyClone, South Logan, UT, USA) and gentamicin (Sigma, St. Louis, MO, USA) at 37 °C in a humidified atmosphere with 5% CO_2_. K-562 cells were grown at RPMI medium (HyClone, South Logan, UT, USA) with the same supplements and condition.

### 2.3. Cell Proliferation Assay

To evaluate the cytotoxicity of compound, cells were seeded at 96 well plates 5 × 10^3^ cells per well. The next day compounds samples were added to the wells. Culture plates were incubated for 1 and 3 days at 37 °C in a humidified atmosphere with 5% CO_2_, then cell proliferation was measured by adding 20 μL of MTS reagent (BioVision, Milpitas, CA, USA) stock solution per well. After 2 h incubation at 37 °C, absorbance at 495 nm was read by Multiskan GO plate spectrophotometer (Thermo Scientific, Waltham, MA, USA). All samples were measured in triplicates.

### 2.4. Cell Cycle Analysis

The distribution of cells in the the G0/G1-, S- and G2/M-phases of the cell cycle was obtained by measuring the relative DNA content in the cells by flow cytometry. 

To perform cell cycle analysis, cells were seeded at 24 well plates, 5 × 10^4^ cells per well. After 24 h incubation, cells were treated with indicated concentration of compounds **4a** and **5a** for 24 h. After that, cells were harvested by trypsyne-EDTA solution and treated by 0.2 mg/mL saponin (Fluka, Rochester, NY, USA), 0.25 mg/mL RNase (Sigma, St. Louis, MO, USA), 0.05 mg/mL propidium iodide (Invitrogen, Waltham, MA, USA) and washed three times. Then, the samples were analyzed by FACSCanto device (Becton Dickinson, Franklin Lakes, NJ, USA). Data processing was performed using the BD FACSDiva 9.0 software.

### 2.5. Annexin V-FITC/DAPI Staining Assay

Cells were seeded in 24 well plates, 5 × 10^4^ cells per well. After 24 h incubation, cells were treated with compound **4a** and **5a** for 72 h. Then cells were washed, harvested by trypsinisation, stained with Annexin V-FITC (BD, Franklin Lakes, NJ, USA) and DAPI (ThermoFisher, Waltham, MA, USA) according to manufactures protocol and analyzed by flow cytometry.

### 2.6. Actin Cytoskeleton Staining

Cells were seeded in Petri dish with cover slips, 2 × 10^5^ cells per dish and incubated for 24 h. After that, cells were treated with **4a** (5 μg/mL) and **5a** (10 μg/mL) compounds for 24 h. Then medium was removed and cells were fixed with 4% paraformaldehyde (Sigma, St. Louis, MO, USA), washed three times with PBS, permeabilizated with Triton-X100 (Sigma, St. Louis, MO, USA), rinsed three times with PBS and stained with rhodamine phalloidine (Invitrogen, Walthman, MA, USA) for 15 min at 37 °C. The preparations were rinsed three times with PBS, and embedded in Fluoroshield medium (Sigma, St. Louis, MO, USA). Intensity of staining of preparations was estimated using an AxioObserver Z1 confocal microscope (Carl Zeiss, Jena, Germany). In each experiment, at least 30 cells were imaged. Images were processed using ImageJ software.

### 2.7. Scratch Test

Cells were seeded in Petri dish, 5 × 10^5^ cells per dish and cultivated until 100% confluence. Then the cell monolayer was scraped in straight line with a 200 μL pipet tip. Full culture media was replaced to serum-free DMEM for inhibition of cell proliferation and cells were treated with **4a** (10μg/mL) and **5a** (20 μg/mL) compounds and incubated 24 h. After that cells were stained with Hoechst 33342 (ThermoScientific, Waltham, MA, USA, 2 μL of 1 mg/mL stock solution to 2 mL of medium) and DIBAC4 (3) (ThermoScientific, Waltham, MA, USA, 2 μL of 1 mg/mL stock solution to 2 mL of medium) for contrasting and evaluated at confocal microcope.

### 2.8. Western Blot Analysis

The protein from whole cell lysates was analyzed by Western blot. Extracts were prepared by lysing cells in RIPA buffer (Sigma-Aldrich, St. Louis, MO, USA). Samples were boiled with Laemmli buffer (BioRad, Hercules, CA, USA), subjected to 10% SDS-PAGE and transferred to nitrocellulose membranes. Then membranes were blocked with 5% non-fat milk in PBS for 1 h at room temperature and incubated in solution containing monoclonal antibodies specific for p53, MDM2 (Cell Signaling Technology, Danvers, MA, USA) and GAPDH (Abcam, Cambridge, UK) overnight at 4 °C. Then membranes were washed with PBS—Tween-20 solution and incubated with HRP-conjugated goat anti-rabbit IgG secondary antibody (Abcam, Cambridge, UK) for 1 h at room temperature. Protein were visualized using the BioRad ECL detection system on the ChemiDoc device (BioRad, Hercules, CA, USA). Processing of obtained images was performed using ImageJ software.

### 2.9. Statistics

Statistical processing of results was performed using MaxStat 3.06 software (MaxStat Software, Germany). All data from the mean ± SE) that three independent experiments were used for measuring the means ± standard error (were compared using the Student′s t-test or nonparametric U-Wilcoxon-Mann-Whitney test. Differences among groups were considered significant at *p* ≤ 0.05.

## 3. Results and Discussion

### 3.1. Synthesis and Structure Elucidation

We have found earlier that containing conjugated O=C–C=N system tryptanthrins (indolo[2,1-*b*]quinazoline-6,12-diones) can form azomethine ylides by interaction with α-amino acids and short peptides. Such formed ylides were used for the synthesis of complex alkaloid-like compounds with spiro-fused indolo[2,1-*b*]quinazoline and cyclopropa[*a*]pyrrolizine or 3-azabicyclo[3.1.0]hexane moieties via one-pot, three-component 1,3-dipolar cycloaddition reactions of various cyclopropenes with in situ generated tryptanthrin-derived azomethine ylides [35]. Later P.R. Likhar et al. have shown that maleimides can be successfully used as dipolarophiles in these 1,3-dipolar cycloaddition reactions to form corresponding spirocyclic product. Even though the formation of only one spiroadduct with all *cis* bridge-protons of pyrrolo[3,4-*a*]pyrrolizine moiety was reported, the configuration of spiroatom was not clarified [40].

We here report that one-pot three-component 1,3-dipolar cycloaddition reactions of various maleimides with in situ generated tryptanthrin-derived azomethine ylide lead to the formation of two diastereomeric products with up to 64% overall isolation yield (Table 1). Both products were effectively separated and isolated individually by PTLC. The ratio of products **4** and **5** was found to be 1.78-1.0 to 1 correspondingly. The major diastereomer in all cases was product **4**, the structures of both isomers were assigned on the basis of NMR spectra analysis. Such ^1^H NMR spectra of adducts **4b** and **5b** exhibit signals for the pyrrole protons at δ 4.05 ppm (d, J = 7.7 Hz, H-C(3a)) and 3.66 ppm (t, J = 7.6 Hz, H-C(8b)) for compound **4b** and δ 4.42 ppm (d, J = 9.8 Hz, H-C(3a)) and 3.63 ppm (dd, J = 9.7 and 6.9 Hz, H-C(8a)) for compound **5b**, and signals for H-C(8a) at δ 4.53–4.61 ppm (m) for compound **4b** and δ 4.58–4.69 ppm (m) for compound **5b**. The shift of the signal of H-C(3a) proton in diastereomer **5b** to a low field [δ 4.05 ppm (**4b**) and δ 4.42 ppm (**5b**)] is due to the deshielding effect induced by the quinazolin fragment of the indolo[2,1-*b*]quinazolin moiety. Plausible pathway for the reaction of maleimide (**2a**) with in situ generated azomethine ylide from tryptanthrin (**1**) and *L*-proline (**3**) is illustrated in Scheme S1 (SI). According to Scheme S1, the formation of four diastereomers is possible, but the only two were found experimentally.

**Figure 1:**
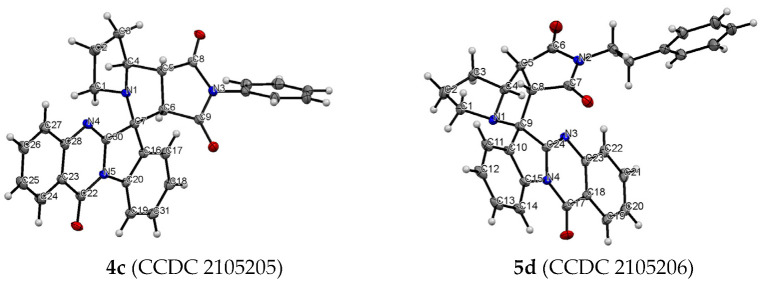
ORTEP representation of the molecular structure of **4c** and **5d**.

The structure and relative configuration of diastereomeric adducts were further unequivocally verified by X-ray crystal analysis of adducts **4c** and **5d** (Figure 1).

### 3.2. Antiproliferative Effect of Synthesized Compounds against Cancer Cell Lines

**Figure 2:**
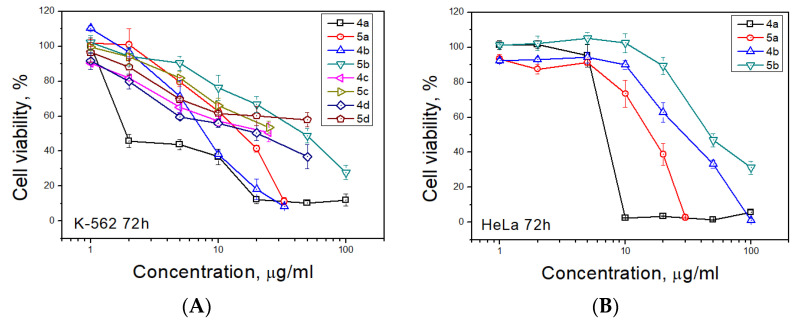
Antiproliferative activity of spiro-fused pyrrolo[3,4-*a*]pyrrolizines and tryptanthrines against K562 (**A**) and HeLa (**B**) cell lines for 72 h.

Antiproliferative activity of synthesized spiro-fused tryptanthrines and pyrrolo[3,4-*a*]pyrrolizines **4** and **5** against human erythroleukemia (K562) and cervical carcinoma (HeLa) cell line was evaluated in vitro by the standard MTS assay for 24 and 72 h. All substances showed no significant cytotoxicity after 24 h of exposure. The results of these investigations after treatment for 72 h are presented in Figure 2. It was found that spiroadducts with all *cis* bridge-protons of pyrrolo[3,4-*a*]pyrrolizine moiety were more active in all cases (compare 4 vs. 5). Replacement of hydrogen atom of pyrrole moiety by either alkyl or aryl group leads to significant decrease in activity of both formed cycloadducts. It was found that among target compounds with spiro-fused tryptanthrin and pyrrolo[3,4-*a*]pyrrolizine moiety only unsubstituted at pyrrole ring cycloadduct **4a** demonstrates significant activity with IC_50_ 1.9 ± 0.2 μg/mL (K562, 72 h), while its diastereomer, **5a**, demonstrates nearly 7 fold lower activity with IC_50_ 14.9 ± 0.5 μg/mL (K562, 72 h). Third most potent ethyl substituted at pyrrole ring cycloadduct **4b** demonstrates intermediate activity with IC_50_ 7.8 ± 0.4 μg/mL (K562, 72 h), while its diastereomer, **5b**, was nearly 6 times weaker with IC_50_ 47.0 ± 0.7μg/mL (K562, 72 h). Since similar results were also found and for HeLa cell lines, all followed tests were performed for cycloadducts **4a** and **5a** only. It should be noted that due to low compound solubility DMSO solutions were added to cultured medium at all the experiments and as negative control the same amount of DMSO was added to control sample (final maximum concentration of DMSO in conducted experiments was 0.35%). IC_50_ values of screened compounds for K562 and HeLa cell lines are given at Table 2 (for IC_50_ given at nM see Table S7).

### 3.3. Cell Death Analysis

**Figure 3:**
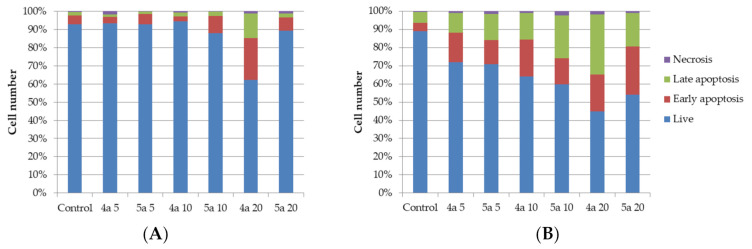
Annexin V-FITC/Propidium iodide (PI) dual staining assay of K562 (**A**) and HeLa (**B**) cells treated with cycloadducts **4a** and **5a** at concentrations 5, 10 and 20 μg/mL using flow cytometry.

The apoptotic effect of compounds **4a** and **5a** was further evaluated by Annexin V-FITC/propidium iodide (AV/PI) dual staining assay to examine the occurrence of phosphatidylserine externalization, which facilitated the detection of live cells (lower left quadrant; AV−/PI−), early apoptotic cells (upper left quadrant; AV+/PI−), late apoptotic cells (upper right quadrant; AV+/PI+) and necrotic cells (lower right quadrant; AV−/PI+) [41]. It was found that HeLa cells were more sensitive for the treatment of both compounds than K562 cell. As shown in Figure 3 and Table 3, the percentage of early apoptotic cells after treatment with compounds **4a** and **5a** increased, respectively, from 5.0% (control) to 23.0 and 9.4% for K562 cell line and from 4.5% (control) to 20.4 and 26.7% for HeLa cell line. The percentage of late apoptotic cells after treatment increased from 1.9% (control) to 13.6 and 2.4% for K562 cell line and from 5.9% (control) to 32.9 and 23.6% for HeLa cell line for compounds **4a** and **5a**, respectively. So the total percentage of apoptotic cells increased for K562 cell line from 6.9% (control) to 36.6 and 11.8% and for HeLa cell line from 10.4% (control) to 53.3 and 43.1% (for compounds **4a** and **5a,** respectively). The percentage of necrotic cells was not significantly changed for both cell lines. 

It was indicated that compounds **4a** and **5a** could induce apoptosis of both HeLa and K562 cells. At the same time, we have demonstrated that compounds **4a** and **5a** lead to inhibition of the growth rate of the cells. 

### 3.4. Cell Cycle Analysis

**Figure 4:**
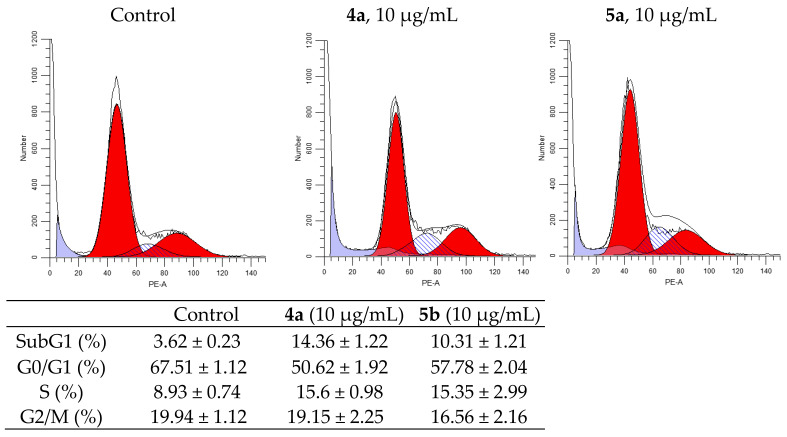
Effect of spiro-fused tryptanthrines and pyrrolo[3,4-*a*]pyrrolizines **4a** and **5a** on the distribution of K562 cells in the cell cycle.

**Figure 5:**
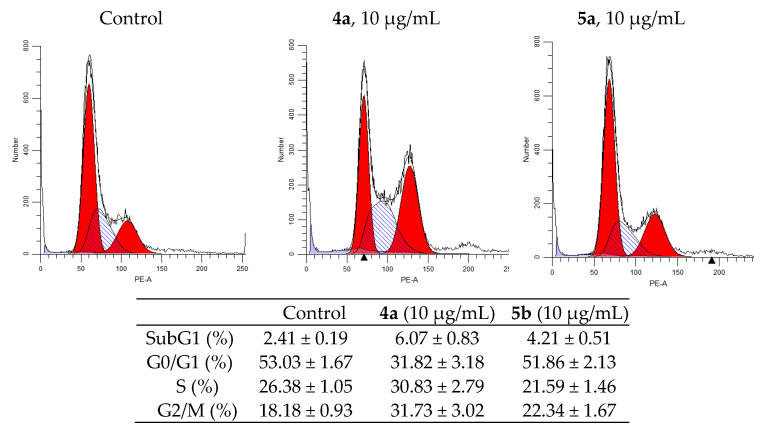
Effect of spiro-fused tryptanthrines and pyrrolo[3,4-*a*]pyrrolizines **4a** and **5a** on the distribution of HeLa cells in the cell cycle.

One of the indicators of the impact of various biologically active substances on the cells is the changes in the cell cycle. In this study, newly synthesized compounds were examined for assessment of the possible distribution of cells in the cell cycle (G0/G1, G2/M and S) using flow cytometry. Figure 4 and Figure 5 shows typical cytometric results and data after processing the results for three replicates of each experiment (for more details see Tables S5 and S6). 

After 24h of incubation of K562 cells, both compounds showed an increase in S-phase from 9.27% in the control cells to 18.22 and 17.11% in the treated cells (for **4a** and **5a**, respectively), and they increased sub-G1 phase from 3.76% to 16.77 and 11.49% (for **4a** and **5a**, respectively). Meanwhile, the percentage of cells in G2/M-phase was nearly the same, while it was decreased in G0/G1 phase to 59.43% for **4a** and to 64.42% for **5a** compared to 70.05% in the untreated cells.

Analysis of the experimental results showed that both compounds stop the HeLa cell cycle in G2/M phase. So, after the impact of compounds **4a** and **5a** at concentration 10 μg/mL for 24h, the percentage of cells in G2/M phase of cycle is 33.78% for compound **4a** and 23.32% for compound **5a** (compared to 18.85% for control). The percentage of cells in the synthetic phase (S) of the cycle is lower for treated with **5b** (22.54%) then untreated cells (26.94%), while for **4a** it was increased (to 32.35%). These findings indicate that the compounds **4a** and **5a** prevent cancer cells from starting DNA division. The strongest cytostatic effect was observed after treatment with **4a** compound. 

**Figure 6:**
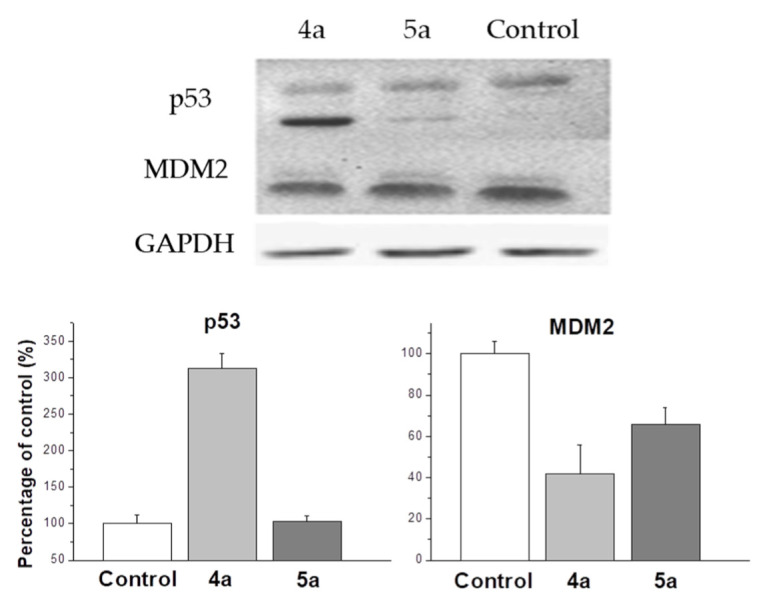
Changes in the relative expression levels of p53 and MDM2 proteins in HeLa cells under the treatment with compounds **4a** and **5a** (at concentration 10 μg/mL).

One of the most studied mechanisms of cell arrest in G2/M phase is the p53 upregulated cell arrest that prevents the transition of cells from G2 to mitosis [42]. Western blot analysis shows an increase in p53 expression up to 310% compared to control under the treatment with **4a** compound (Figure 6). MDM2 protein is negative regulator of p53 tumor suppressor. Thus, although compound **5a** does not affect the expression of p53, a decrease in the expression of MDM2 causes a cell cycle arrest in the G2/M phase, but with less efficiency than **4a**, inducing both the activation of the expression of p53 and a decrease in the expression of MDM2. In our work, it was shown that **4a** and **5a** compounds stop the cell cycle at the G2/M phase, while in other studies it was shown that cell cycle arrest can occur in both the G0/G1 and G2/M phases [43,44]. A significant dependence of the efficiency of inhibition of the cell cycle depending on the conformation of the spiro-fused heterocycle has been shown. In addition, the results of western blot analysis suggest the presence of various mechanisms that trigger the arrest of the cell cycle in the G2/M phase. Thus, exposure to compound **4a** triggers an arrest associated with an increase in p53, while **5a** does not affect the content of p53 in the cell lysate.

### 3.5. Inhibition of Cell Motility Evaluated by Scratch-Test

Cell motility is a fundamental and ancient cellular behaviour that contributes to metastasis [45]. A major challenge in understanding metastatic tumour spread in patients is that the process cannot be observed or manipulated directly. Scratch-test is a simple model to assess the impact of different effects on cell motility and potential metastasis.

**Figure 7:**
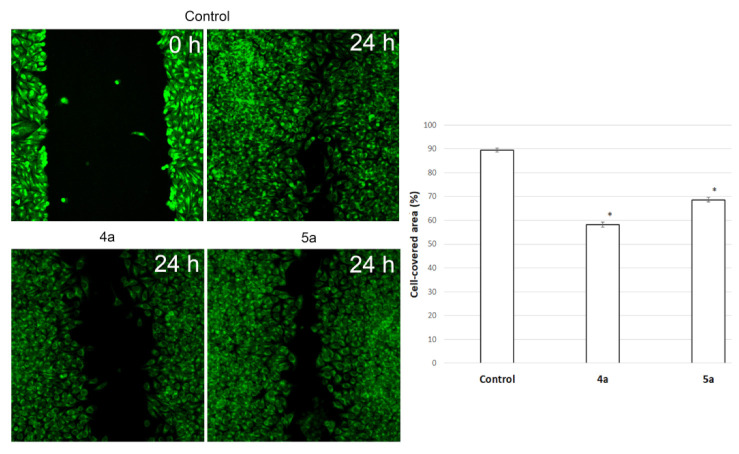
Microscopic images of the HeLa cells wound area in the scratch assay and wound area (%) in the scratch assay after 24 h incubation post spiro-fused cycloadducts **4a** and **5a** treatment at concentration 10 μg/mL. * *p* value < 0.05.

To assess the potential ability of compounds to inhibit metastasis associated with cell motility, a Scratch-test was performed on human cervical carcinoma (HeLa) cell line. We used Hoechst 33,342, membrane penetrable nuclear dye and DiBAC_4_(3)—very bright and easy to use hydrophilic anion dye for fast and non-toxic visualization of cells. The result is shown in Figure 7. Nontreated HeLa cells filled the scratchad strip at 89.4 ± 0.8% while under treatment with compounds **4a** (10 μg/mL) and **5a** (20 μg/mL) cells fill 58.2 ± 1% and 68.6 ± 0.9% of scratched strip, respectively. Hela cells lose their ability to move under treatment and do not fill the scratched strip. 

### 3.6. Actine Cytoskeleton Changes

It is known that actin plays an important role in vital cellular processes, providing a number of functions such as cell adhesion, migration and morphogenesis [46,47]. Actin cytoskeleton may serve as an additional target of antitumor chemotherapy [48,49]. Tumor transformation causes reorganization of the actin cytoskeleton, leading to change in cell motility. Correlation between the actin assembly and organization and increased migration activity of tumor cells was observed [50,51]. The structural features of actin organization can serve as criteria for assessing the metastasis potential of tumor cells [52]. HeLa cell line is widely used to study the structure of the actin cytoskeleton and was characterized by presence of filopodia and actin stress fibers [53].

**Figure 8:**
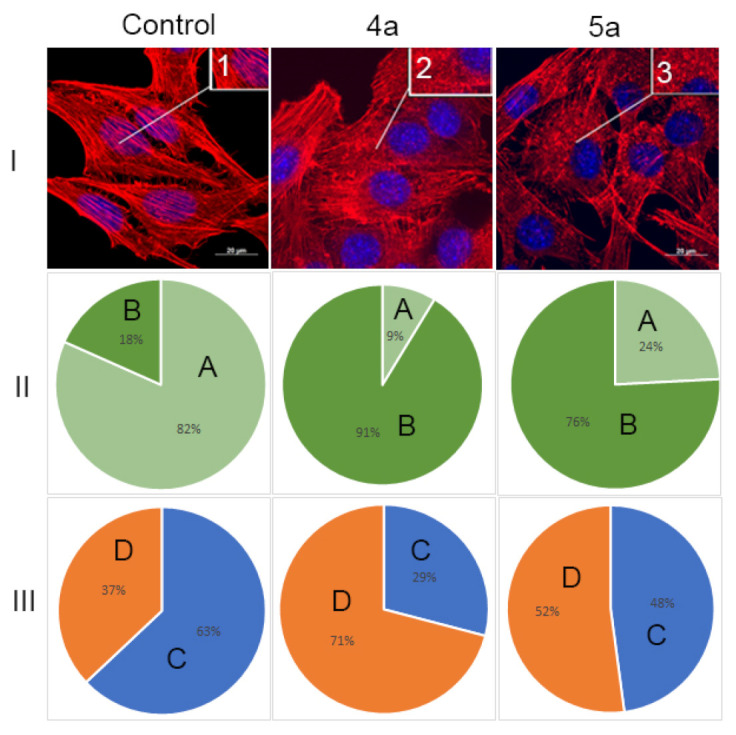
State of actin cytoskeleton of HeLa cells after treatment with spiro-fused tryptanthrines and pyrrolo[3,4-*a*]pyrrolizines **4a** and **5a**. (**I**): Images demonstrate the different stages of cell actin cytoskeleton. (**II**): Pie charts demonstrate percentage of cells with normal stress fibers (A) and disassembled stress fibers (B). (**III**): Pie charts demonstrate percentage of cells with filopodia-like deformations (C), and without filopodia-like deformations (D). Inserts: 1—stress fibers; 2—disassembled stress fibers.

Therefore, in this study, the structure of the actin cytoskeleton of HeLa cells was assessed by the availability of stress fibers and the presence of filopodia-like protrusions after the impact of compounds **4a** and **5a** (Figure 8). It was found using confocal microscopy that treatment with cycloadducts **4a** and **5a** at concentration 10 μg/mL led to significant changes of the actin cytoskeleton structure of tumor cells leading to the disappearance of stress fibers and changes in the number of filopodia-like deformations. Such treatment with compounds **4a** and **5a** led to decrease in the number of cells with stress fibers from 82% to 9% and 24%, respectively. Similarly the number of cells with filopodia-like structures decreased from 63% in control sample to 29% and 48% in cells treated with either **4a** or **5a**, respectively. 

We can assume that compounds **4a** and **5a** combine both anti-proliferative and anti-migratory action. We have shown that the action of the compounds leads to a change in the structure of the actin cytoskeleton, including a decrease in the number of filopodia-like deformations. This may indicate a decrease in cell motility. Together with the scratch test data, we can assume that compounds **4a** and **5a** also affect cell motility. In addition, compounds active in anti-proliferation against cancer cells were seen to possess better anti-migration ability [54].

## 4. Conclusions

In this study, we have established the ability of heterocyclic compounds containing spiro-fused pyrrolo[3,4-*a*]pyrrolizine and tryptanthrin framework to reduce the viability of both human erythroleukemia (K562) and human cervical carcinoma (HeLa) cell lines. We have found that spiroadducts with all *cis* bridge-protons of pyrrolo[3,4-*a*]pyrrolizine moiety were more active in all cases. Replacement of hydrogen atom of pyrrole moiety by either alkyl or aryl group leads to significant decrease in activity of both formed cycloadducts. Such it was found that among target compounds only unsubstituted at pyrrole ring cycloadduct **4a** demonstrates significant activity with IC_50_ 1.9 ± 0.2 μg/mL (K562, 72 h), while its diastereomer, **5a**, demonstrates nearly 7 fold lower activity with IC_50_ 14.9 ± 0.5 μg/mL (K562, 72 h). Third most potent ethyl substituted at pyrrole ring cycloadduct **4b** demonstrates intermediate activity with IC_50_ 7.76 ± 0.9 μg/mL (K562, 72 h) and its diastereomer, **5b**, was nearly 6 times weaker with IC_50_ 46.67 ± 3.7 μg/mL (K562, 72 h). Both cycloadducts **4a** and **5a** were shown to downregulate growth of Hela cells, as well as arrest the cell cycle in the G2/M phase, lead to a decrease in the number of cells with normal stress fibers and filopodia-like deformations, induce apoptosis and decrease cell motility. All obtained data make it possible to assume that spiro-fused pyrrolo[3,4-a]pyrrolizine and tryptanthrin framework may be considered as a new promising pharmacophore unit for further screenings.

## Figures and Tables

**Table 1:**
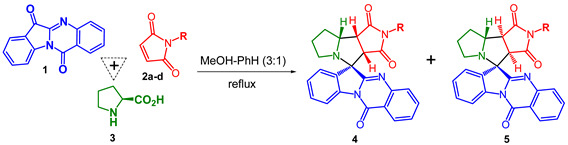
One-pot three-component reactions of tryptanthrin **1**, maleimides **2a-d** and *L*-proline **3**.

Entry	Maleimide	Substituent R	Ratio 4:5	Yield, % ^1^	
				Product 4	Product 5
1	**2a**	H	1.15:1.0	34	30
2	**2b**	Et	1.25:1.0	29	23
3	**2c**	Ph	1.0:1.0	7	7
4	**2d**	CH_2_CH_2_Ph	1.78:1.0	20	11

^1^ Isolated yield.

**Table 2:** IC_50_ values (μg/mL) of screened compounds for K562 and HeLa cell line.

	4a	5a	4b	5b	4c	5c	4d	5d
**K562**	1.9 ± 0.2	14.9 ± 0.5	7.8 ± 0.4	47.0 ± 0.7	>50	>50	20.5 ± 0.7	>50
**HeLa**	6.9 ± 0.4	16.2 ± 0.5	29.7 ± 0.7	46.8 ± 1.1	-	-	-	-

**Table 3:** Annexin V-FITC/Propidium iodide (PI) dual staining assay of K562 and HeLa cells treated with cycloadducts **4a** and **5a** at concentrations 5, 10 and 20 μg/mL using flow cytometry.

**K562**	**Control**	**4a**			**5a**		
		**5 μg/mL**	**10 μg/mL**	**20 μg/mL**	**5 μg/mL**	**10 μg/mL**	**20 μg/mL**
Live	92.8 ± 1.4	93.3 ± 0.7	94.3 ± 0.6	62.2 ± 3.7	92.8 ± 0.1	88.0 ± 0.2	89.3 ± 0.1
Early apoptosis	5.0 ± 2.0	3.7 ± 1.0	2.9 ± 0.5	23.0 ± 3.0	5.7 ± 0.3	9.4 ± 0.1	7.5 ± 0.2
Late apoptosis	1.9 ± 0.3	1.4 ± 0.1	2.0 ± 0.3	13.6 ± 1.8	1.1 ± 0.1	2.4 ± 0.2	1.9 ± 0.5
Necrosis	0.3 ± 0.3	1.6 ± 0.2	0.8 ± 0.2	1.2 ± 0.4	0.2 ± 0.1	0.2 ± 0.1	1.3 ± 0.4
**HeLa**	**Control**	**4a**			**5a**		
		**5 μg/mL**	**10 μg/mL**	**20 μg/mL**	**5 μg/mL**	**10 μg/mL**	**20 μg/mL**
Live	89.0 ± 0.7	72.0 ± 1.2	64.0 ± 1.1	44.9 ± 1.8	70.8 ± 0.4	59.7 ± 1.3	53.9 ± 0.2
Early apoptosis	4.5 ± 0.7	16.3 ± 2.1	20.3 ± 2.4	20.4 ± 1.8	13.3 ± 0.4	14.3 ± 1.0	26.7 ± 0.3
Late apoptosis	5.9 ± 0.4	10.8 ± 0.8	14.8 ± 1.3	32.9 ± 0.3	14.3 ± 0.1	23.6 ± 0.5	18.4 ± 0.2
Necrosis	0.5 ± 0.3	0.9 ± 0.3	0.9 ± 0.3	1.8 ± 0.1	1.6 ± 0.1	2.4 ± 0.2	0.9 ± 0.1

## Data Availability

The data is available in the Supplementary Materials.

## Supplementary Materials

The following are available online at https://www.mdpi.com/article/10.3390/ijms222111997/s1.
