# Supplementary material for: Identification of Spiro-Fused Pyrrolo[3,4-a]pyrrolizines and Tryptanthrines as Potential Antitumor Agents: Synthesis and In Vitro Evaluation
Source: Int J Mol Sci. 2021 Nov 5;22(21):11997. doi: 10.3390/ijms222111997 (PMC8584944; doi:10.3390/ijms222111997)
Supplement: Supplementary file 1 [file ijms-22-11997-s001.zip › ijms-1425344-supplementary.pdf]

Supporting information

Identification of Spiro-Fused Pyrrolo[3,4-*a*]pyrrolizines and Tryptanthrines as Potential Antitumor Agents: Synthesis and *in vitro* Evaluation

Diana. K. Latypova [1], Stanislav V. Shmakov [1], Sofya A. Pechkovskaya [2], Alexander S. Filatov [3], Alexander V. Stepakov [3,4], Nickolay A. Knyazev [2,5,*] and Vitali M. Boitsov [1*]

[1] Saint Petersburg National Research Academic University of the Russian Academy of Sciences, Saint Petersburg, Russia
[2] Institute of Cytology, Russian Academy of Sciences, Saint Petersburg, Russia
[3] Saint Petersburg State University, Saint Petersburg, Russia
[4] Saint Petersburg State Institute of Technology, Saint Petersburg, Russian Federation
[5] Saint-Petersburg Clinical Scientific and Practical Center for Specialized Types of Medical Care (Oncological), Saint-Petersburg, Russia
* Correspondence: N.A.K.: nickolayknz@gmail.com; V.M.B.: bovitali@yandex.ru

Table of contents

1. Plausible reaction mechanism for the formation of compounds **4** and **5** **S2**

2. Copies of ^{1}H and ^{13}C NMR spectra of compounds **4** and **5** **S3**

3. 2D NMR spectra of compound **4b** and **5b** **S10**

4. Bioassay details **S11**

1. Plausible reaction mechanism for the formation of compounds 4 and 5

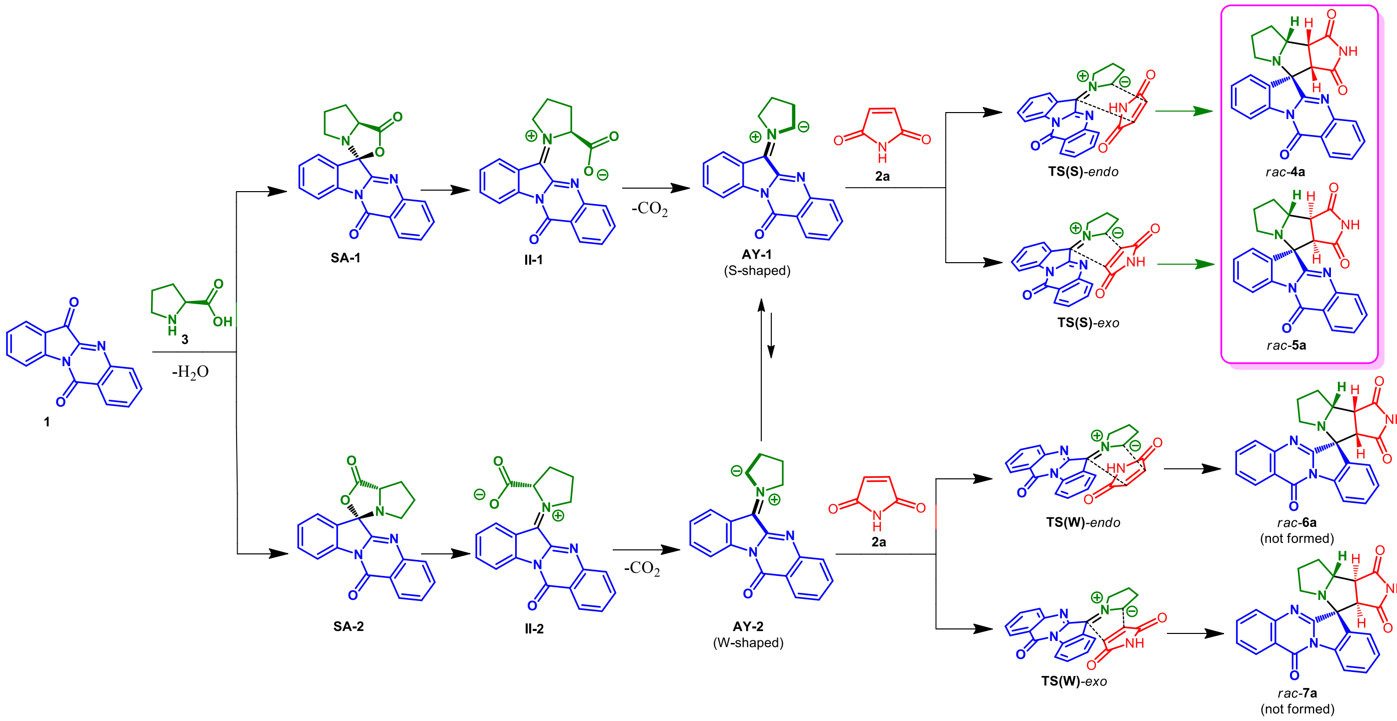

Scheme S1. Plausible reaction mechanism for the formation of compounds **4** and **5**

Comment: In the first stage, spiro-pyrrolooxazoles **SA-1** and **SA-2** are formed from tryptanthrine (**1**) and L-proline (**3**). Further, **SA-1** and **SA-2** through the stage of iminium intermediates **II-1** and **II-2** are converted into *S*- and *W*-shaped azomethine ylides **AY-1** and **AY-2**, respectively. At the last stage, 1,3-dipolar cycloaddition occurs between azomethine ylides and maleimide (**2a**). As a result of this reaction, it is possible to identify only compounds **4a** and **5a** - the products of the interaction of S-ylide **AY-1** with maleimide (**2a**).

2. Copies of ^{1}H and ^{13}C NMR spectra of compounds 4 and 5

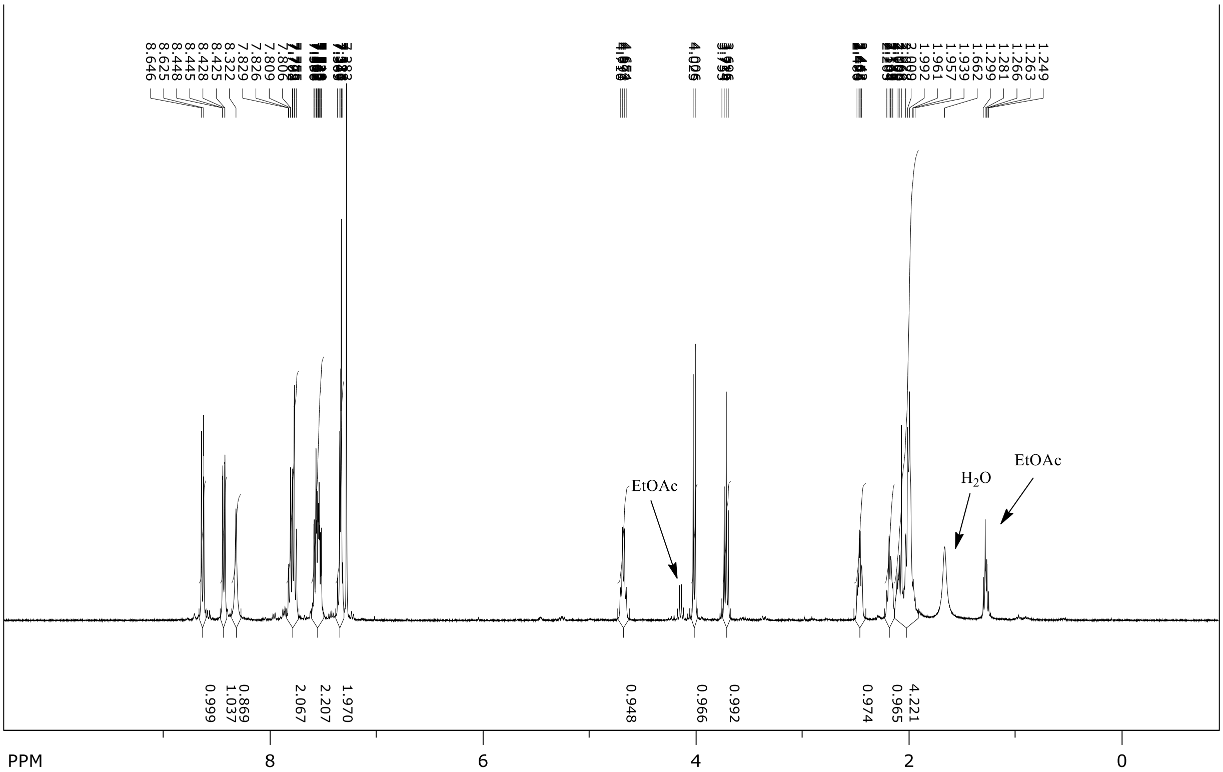

Figure S1. ^{1}H NMR spectrum of compound **4a** ($CDCl_3$, 400 MHz)

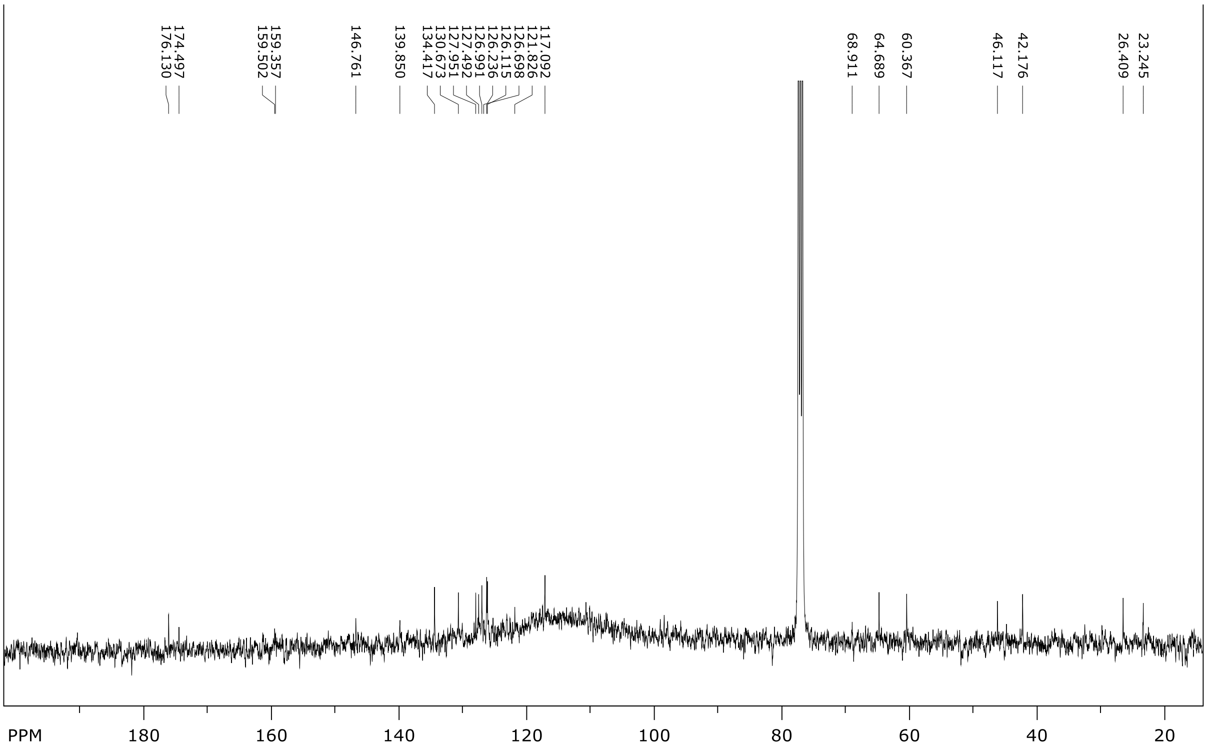

Figure S2. ^{13}C NMR spectrum of compound **4a** ($CDCl_3$, 101 MHz)

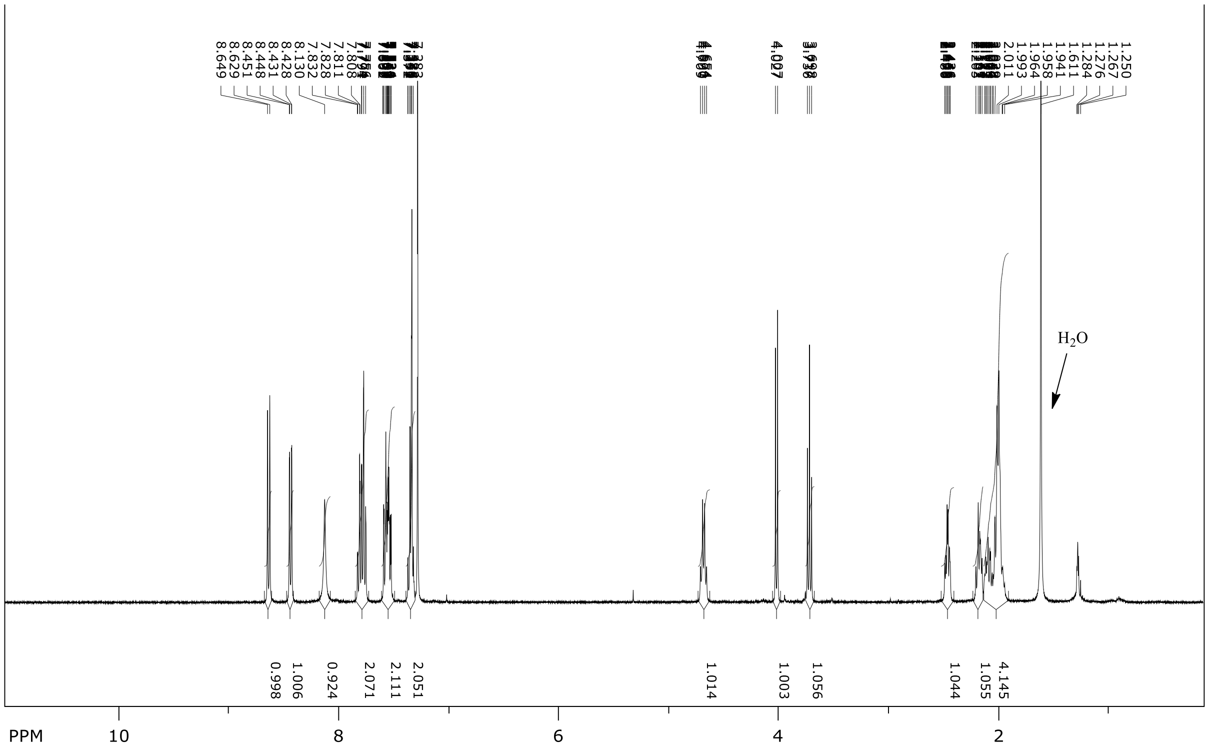

Figure S3. ^{1}H NMR spectrum of compound **5a** ($CDCl_3$, 400 MHz)

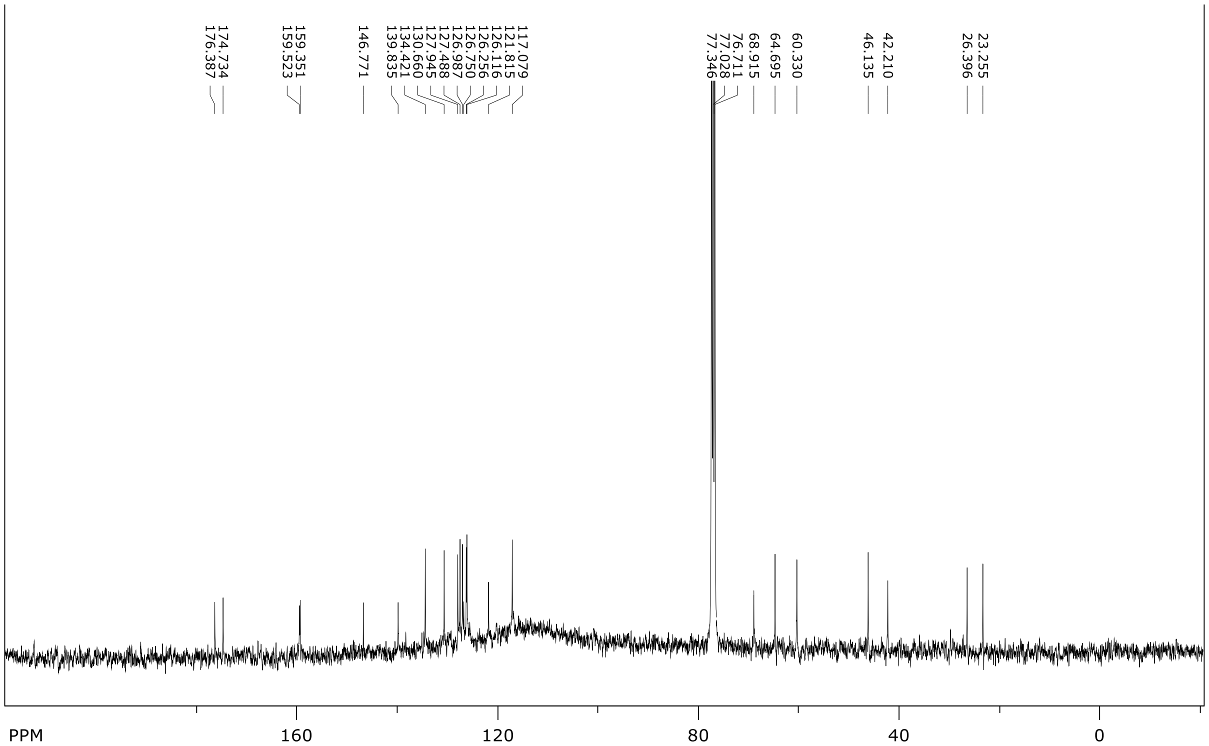

Figure S4. ^{13}C NMR spectrum of compound **5a** ($CDCl_3$, 101 MHz)

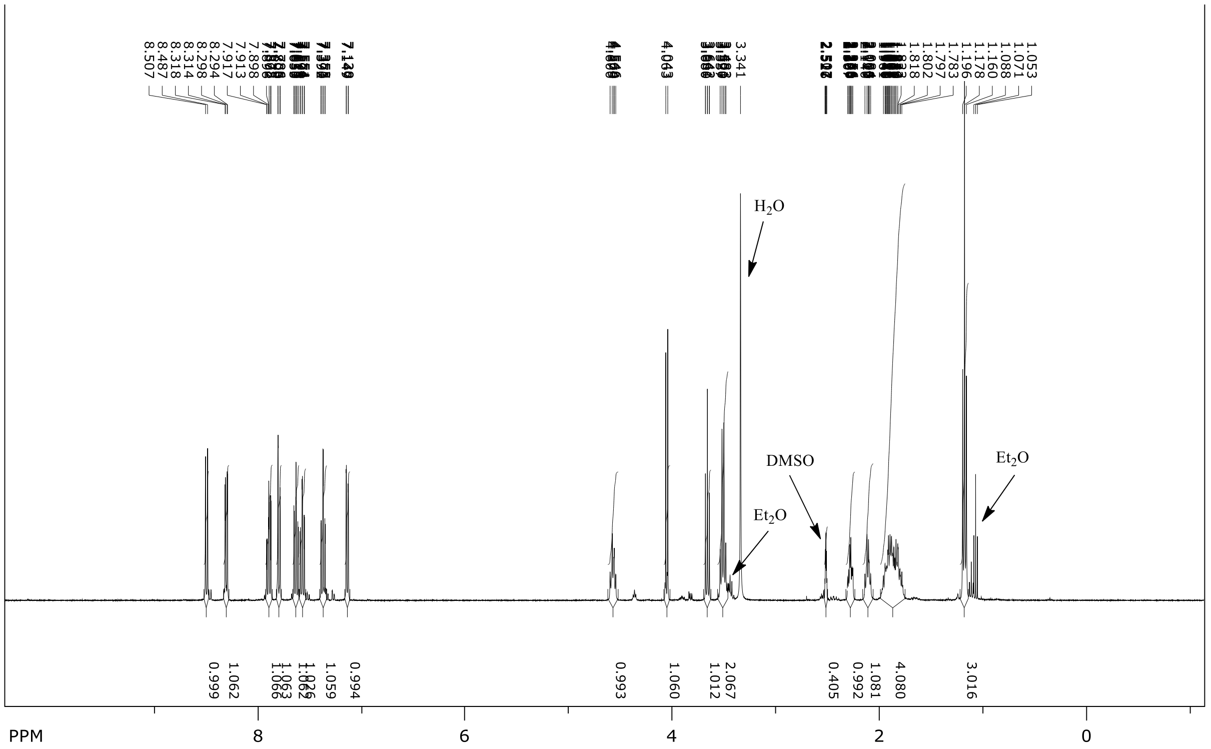

Figure S5. ^{1}H NMR spectrum of compound **4b** (DMSO-*d6*, 400 MHz)

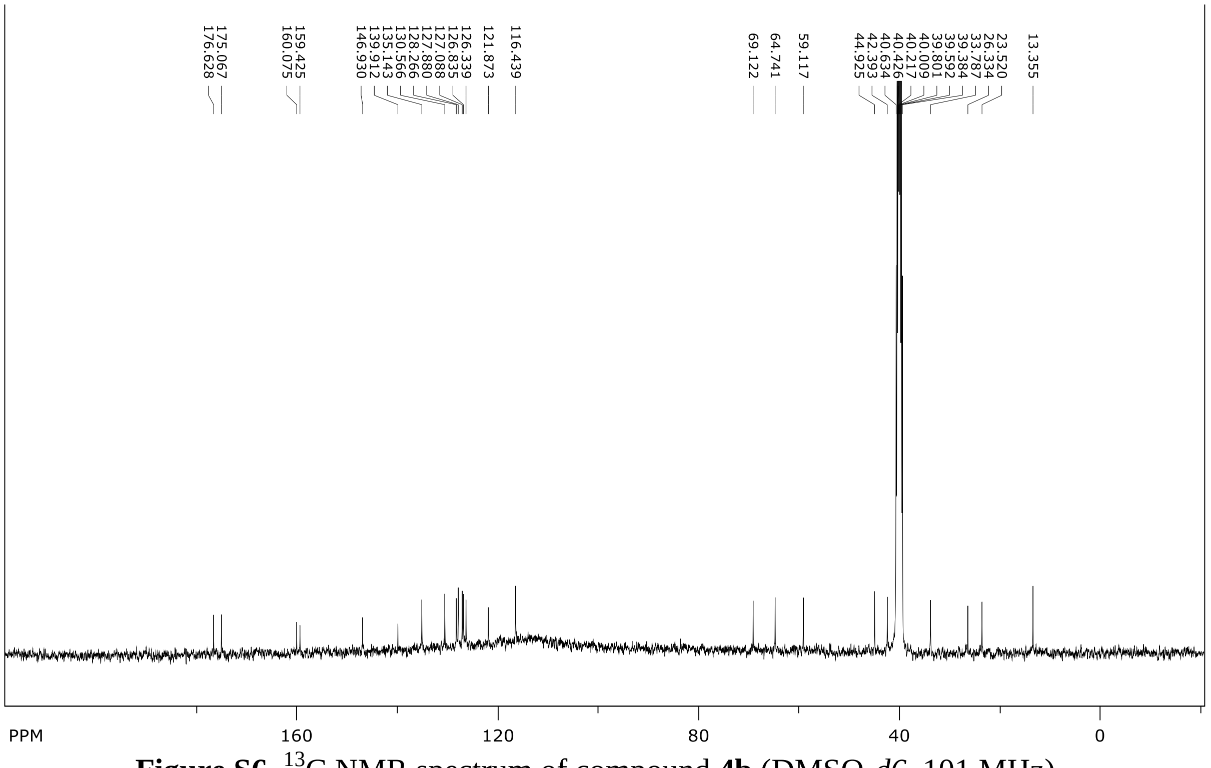

Figure S6. ^{13}C NMR spectrum of compound **4b** (DMSO-*d6*, 101 MHz)

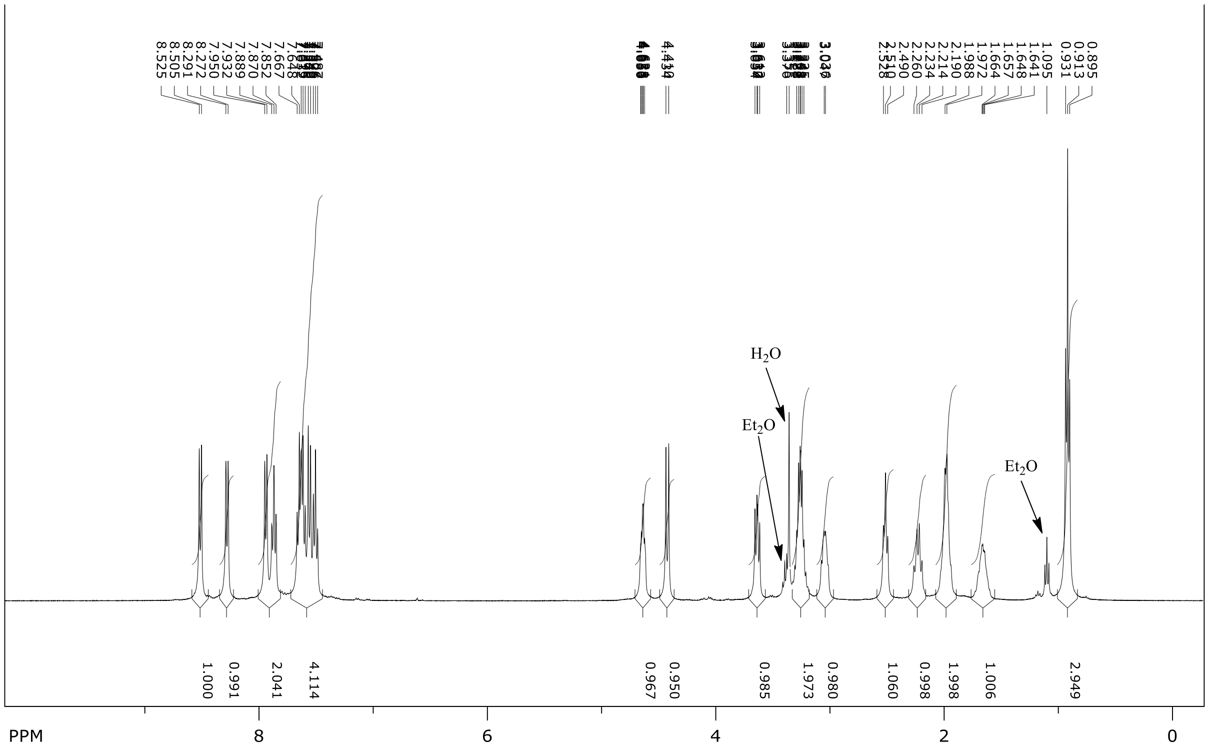

Figure S7. ^{1}H NMR spectrum of compound **5b** (DMSO-*d6*, 400 MHz)

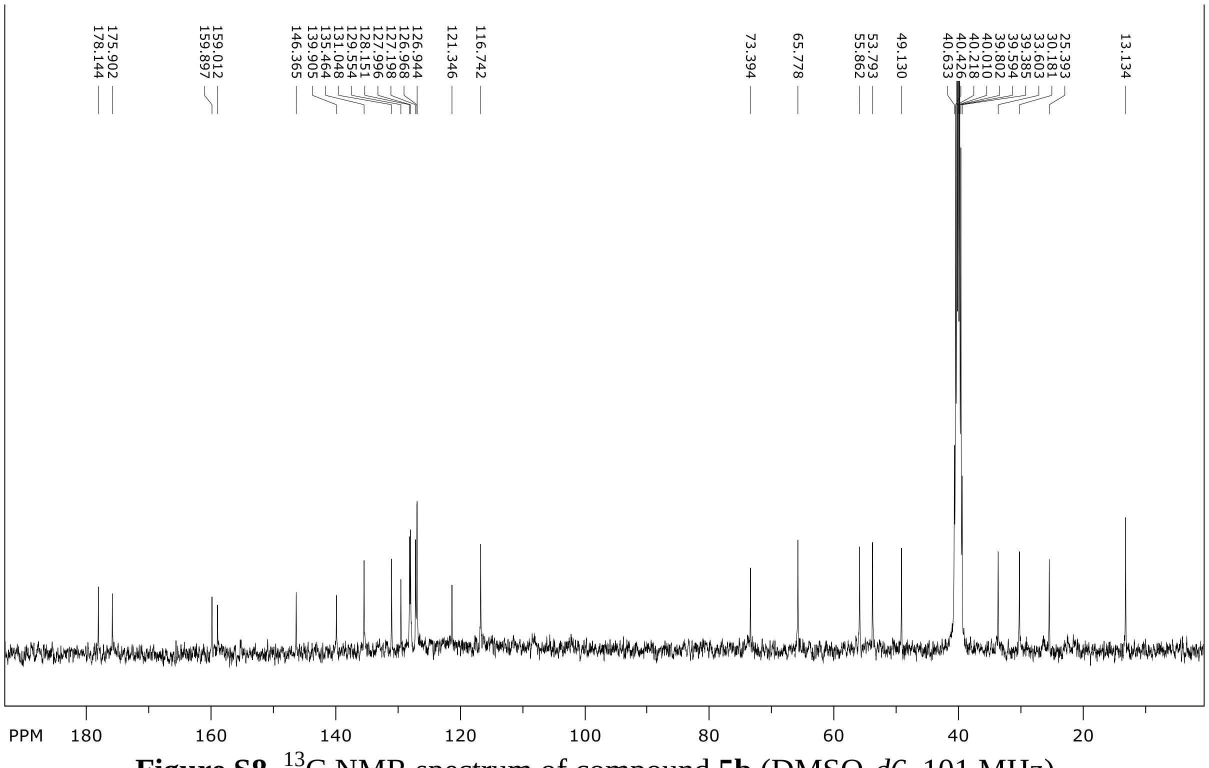

Figure S8. ^{13}C NMR spectrum of compound **5b** (DMSO-*d6*, 101 MHz)

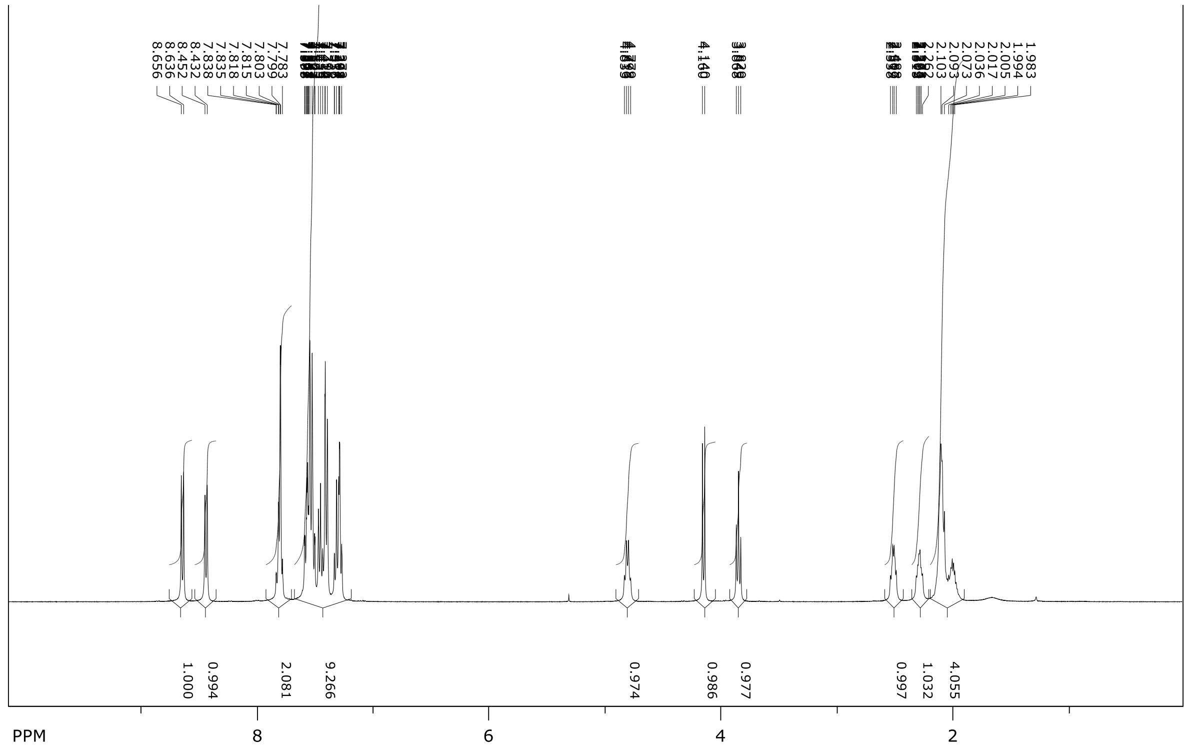

Figure S9. ^{1}H NMR spectrum of compound **4c** ($CDCl_3$, 400 MHz)

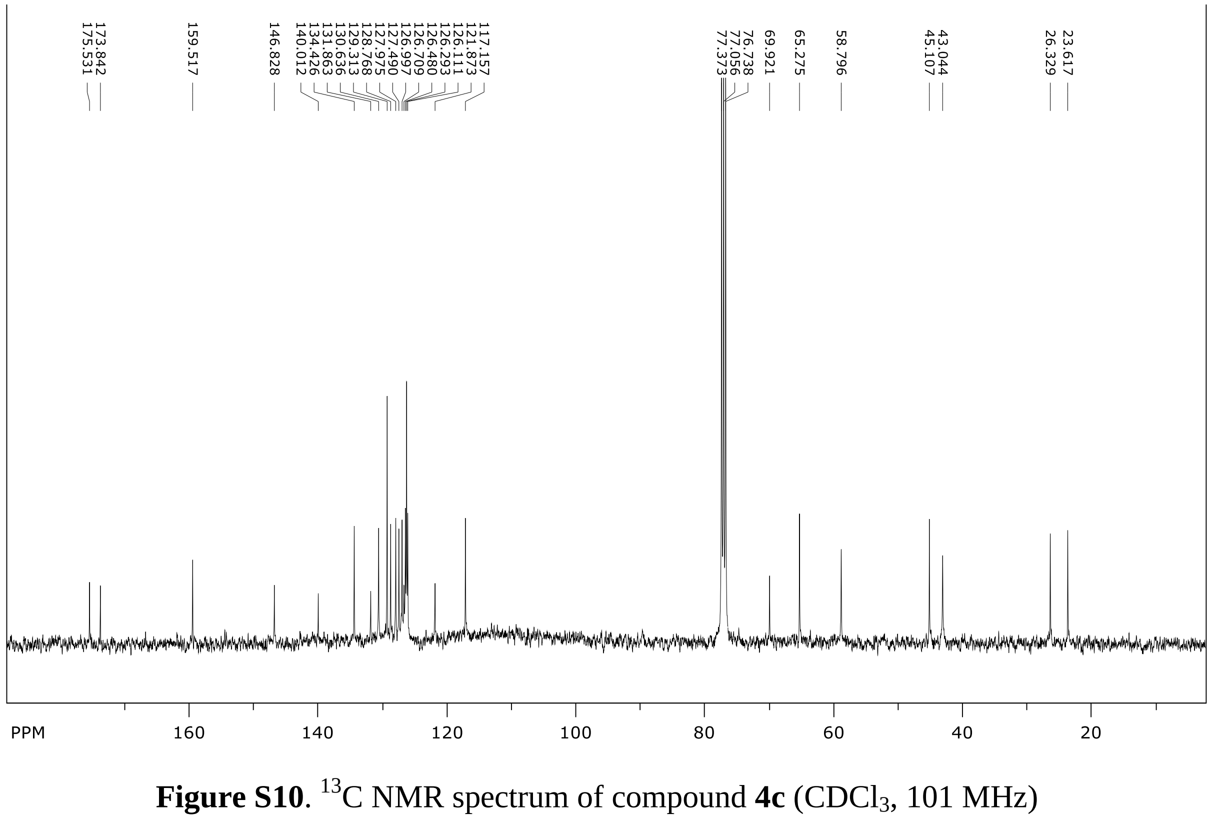

Figure S10. ^{13}C NMR spectrum of compound **4c** ($CDCl_3$, 101 MHz)

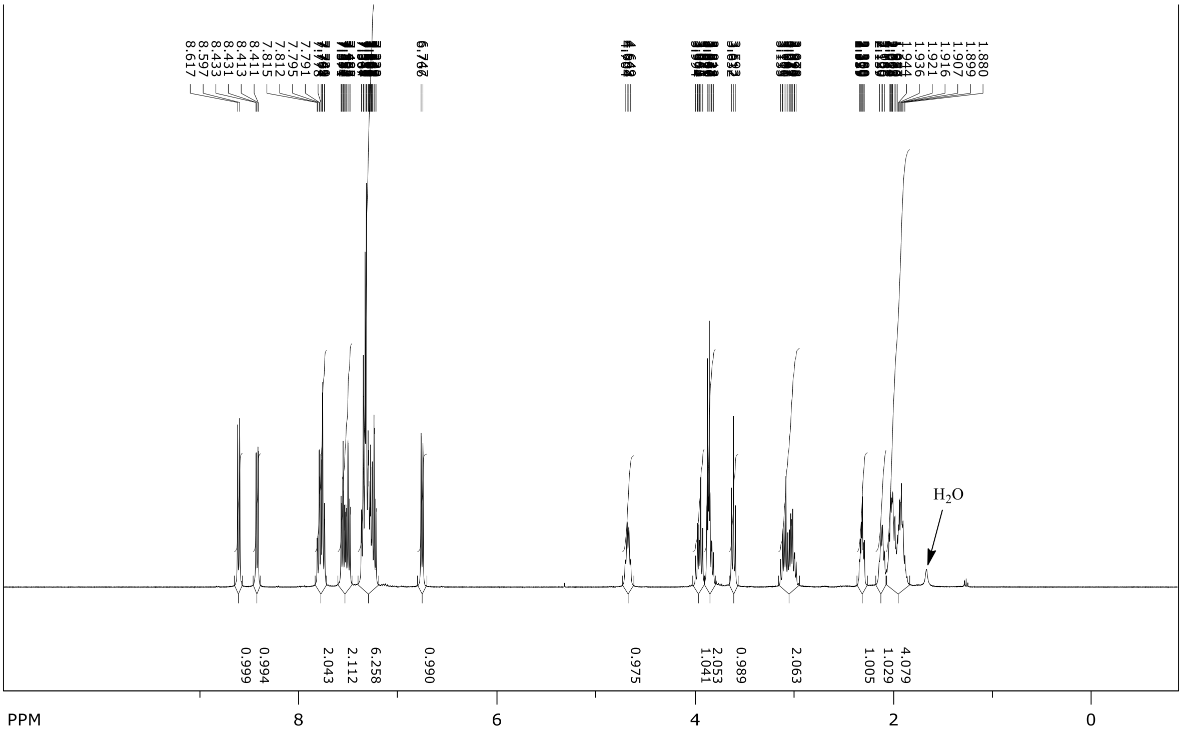

Figure S11. ^{1}H NMR spectrum of compound **4d** ($CDCl_3$, 400 MHz)

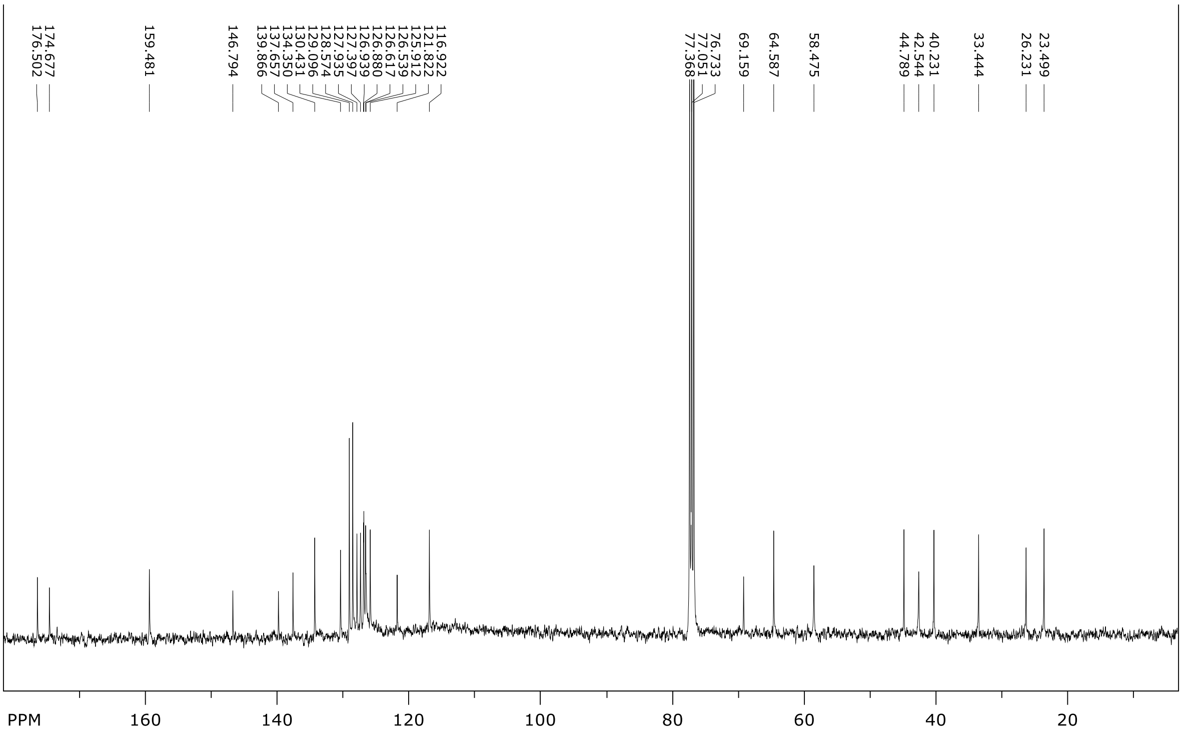

Figure S12. ^{13}C NMR spectrum of compound **4d** ($CDCl_3$, 101 MHz)

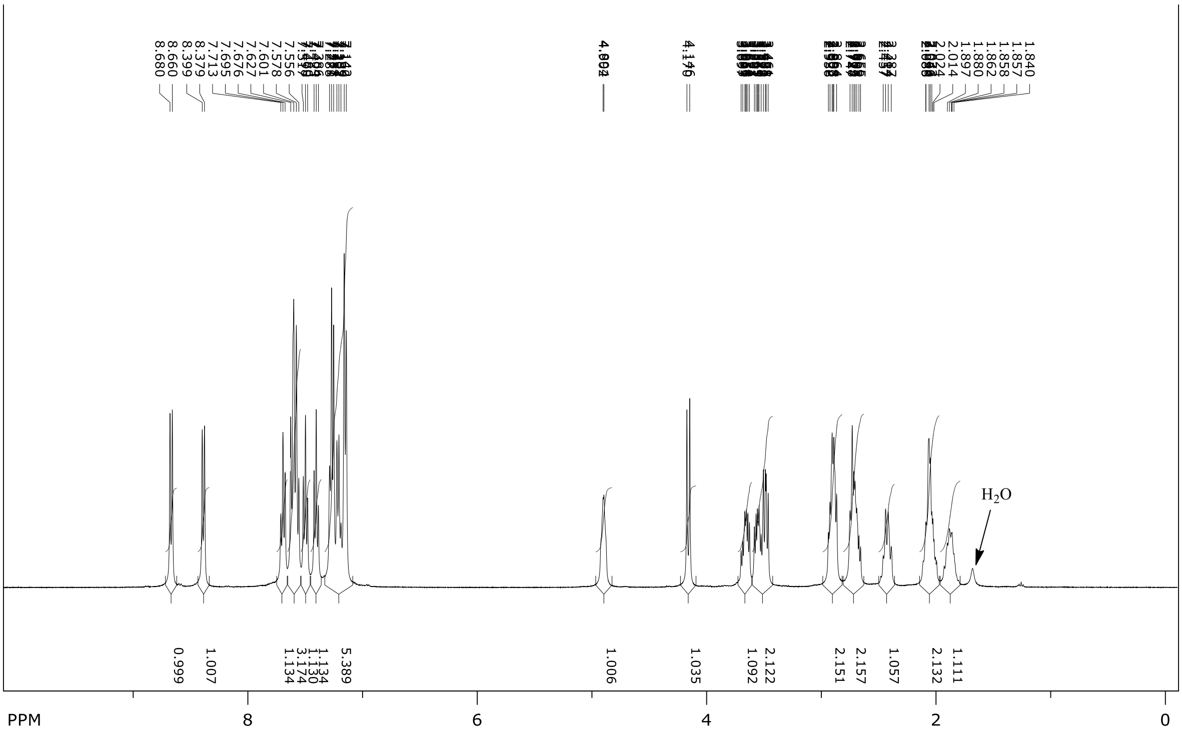

Figure S13. ^{1}H NMR spectrum of compound **5d** ($CDCl_3$, 400 MHz)

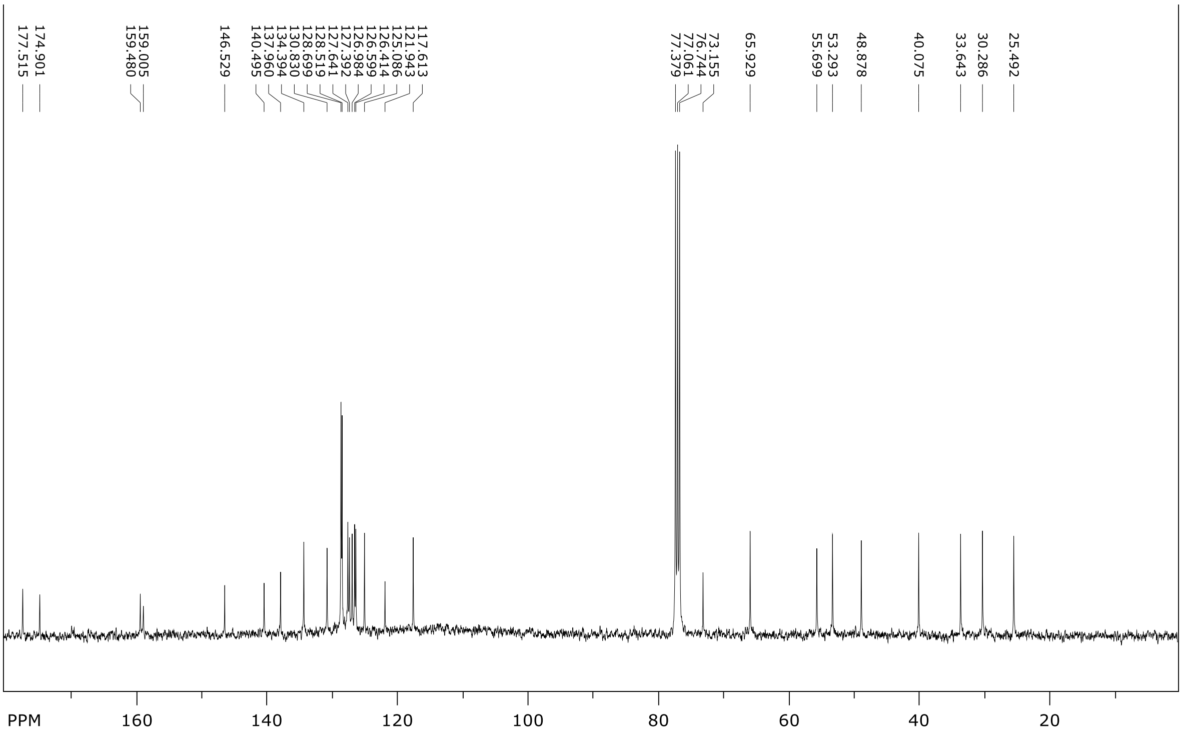

Figure S14. ^{13}C NMR spectrum of compound **5d** ($CDCl_3$, 101 MHz)

3. 2D NMR spectra of compound 4b and 5b

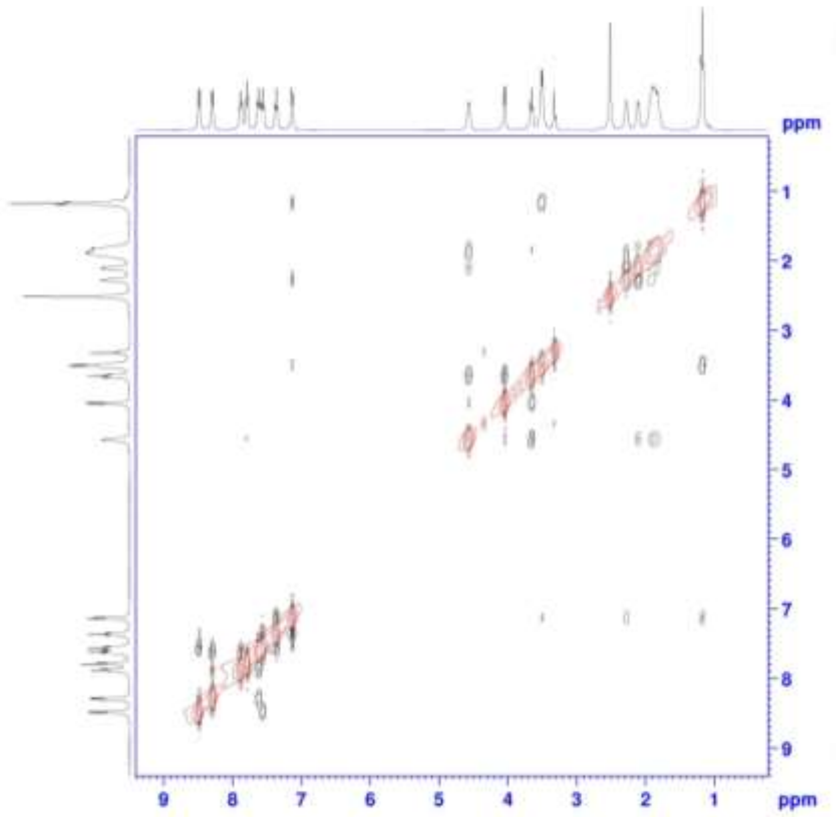

Figure S15. NOESY spectrum of compound **4b** (DMSO-*d6*, 400 MHz)

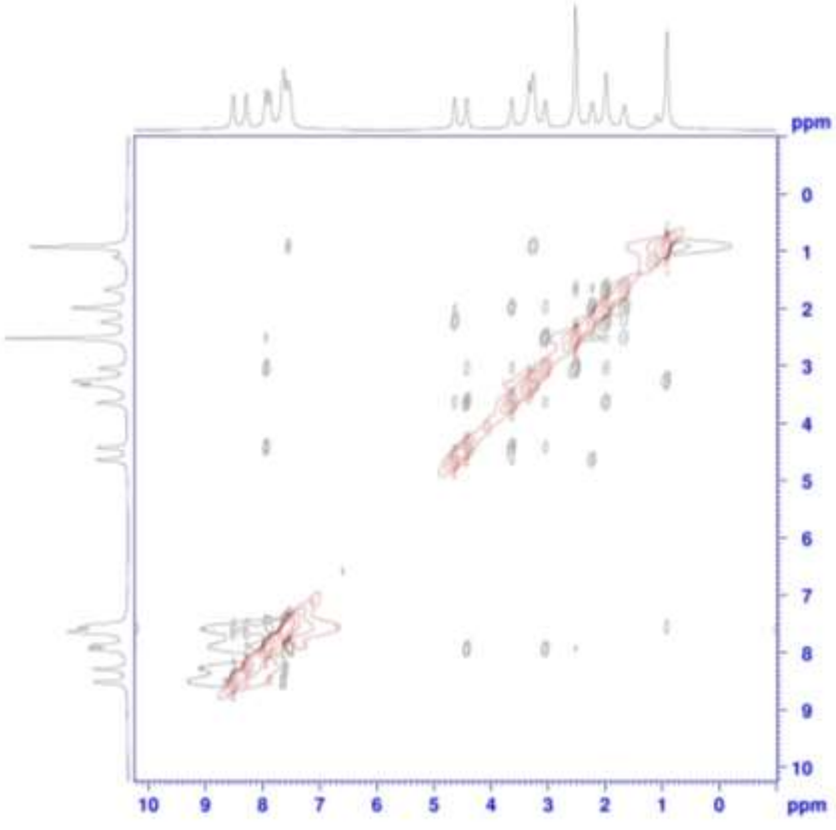

Figure S16. NOESY spectrum of compound **5b** (DMSO-*d6*, 400 MHz)

4. Bioassay details

Table S1. K562 cells viability after the treatment for 72 h

Compound	Cell viability							
	1 μg/ml	2 μg/ml	5 μg/ml	10 μg/ml	20 μg/ml	30 μg/ml	50 μg/ml	100 μg/ml
**4a**	100.4±4.7	45.8±3.9	43.7±3.1	36.7±4.6	12.1±2.2	n.d.[1]	10.2±1.4	12.0±3.4
**5a**	101.9±1.6	101.0±8.0	79.6±2.2	62.8±3.7	41.4±1.7	11.3±1.8	n.d.	n.d.
**4b**	110.3±1.5	96.5±2.6	71.3±9.2	38±2.1	18.1±5.8	8.3±1.2	n.d.	n.d.
**5b**	102.1±4.0	94.2±5.5	90.3±4.1	76.3±7.2	66.7±4.6	n.d.	48.8±4.2	27.8±4.2
**4c**	90.4±0.9	82±1.5	65.1±3.4	57.3±2.6	50.8±5.5	n.d.	n.d.	n.d.
**5c**	99.6±1.8	93.9±4.1	81.9±3.8	66.2±4.1	53.7±3.4	n.d.	n.d.	n.d.
**4d**	91.7±4.8	79.9±4.2	59.6±1.3	56.0±2.5	50.4±4.3	n.d.	36.8±6.8	n.d.
**5d**	96.5±5.6	88.2±4.7	69.6±0.6	61.5±0.6	60.1±5.5	n.d.	58±4.0	n.d.

[1] Not determined.

Table S2. HeLa cells viability after the treatment for 72 h

Compound	Cell viability							
	1 μg/ml	2 μg/ml	5 μg/ml	10 μg/ml	20 μg/ml	30 μg/ml	50 μg/ml	100 μg/ml
**4a**	101.4±2.1	101.6±1.9	95.2±5.4	2.5±0.1	3.2±0.1	n.d.[1]	1.4±0.3	5.6±1.1
**5a**	93.4±2.4	87.6±2.5	91.5±1.6	73.5±6.6	38.9±5.3	2.7±0.2	n.d.	n.d.
**4b**	92.2±1.4	92.9±1.3	94.2±3.0	90±2.5	62.8±5.5	n.d.	33.1±2.3	1.1±1
**5b**	101.1±0.8	102.3±4.1	105.1±3.3	102.5±5.4	89.5±4.9	n.d.	46.9±3.8	31.2±3.8

[1] Not determined.

Table S3. Annexin V-FITC/Propidium iodide (PI) dual staining assay of K562 cells treated with cycloadducts **4a** and **5a** at concentrations 5, 10 and 20 μg/ml using flow cytometry

Compound	Concentration, μg/ml			Control
	5	10	20	
**4a**	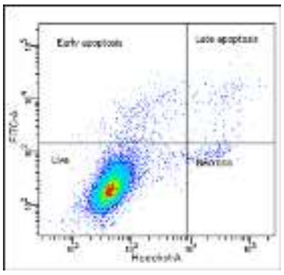	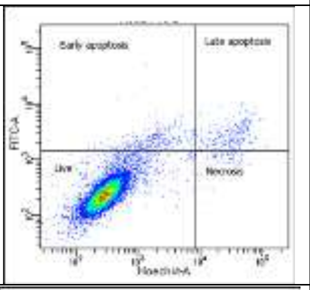	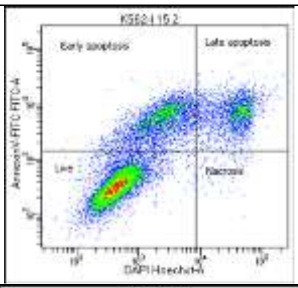	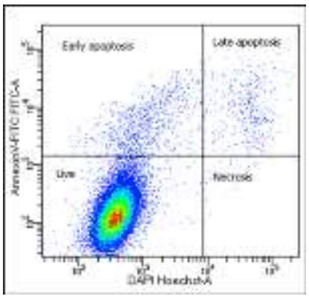
**5a**	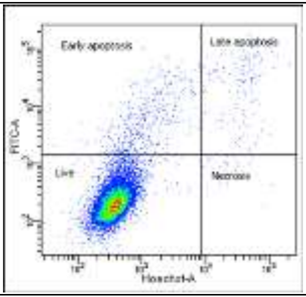	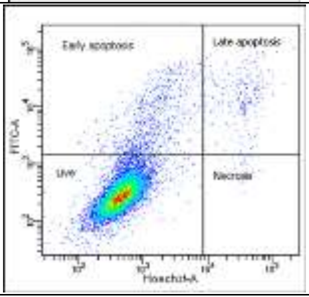	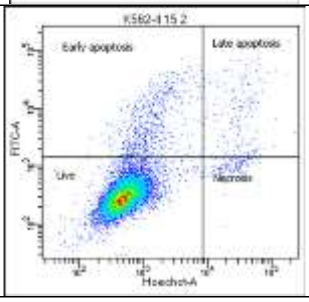	

Table S4. Annexin V-FITC/Propidium iodide (PI) dual staining assay of HeLa cells treated with cycloadducts **4a** and **5a** at concentrations 5, 10 and 20 μg/ml using flow cytometry

Compound	Concentration, μg/ml			Control
	5	10	20	
**4a**	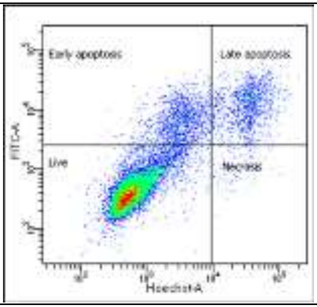	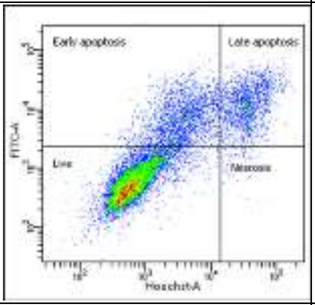	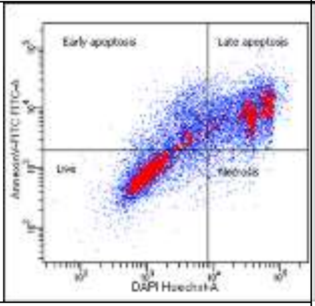	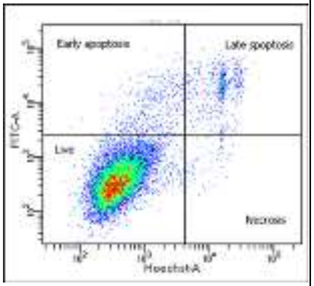
**5a**	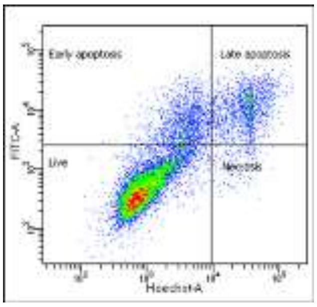	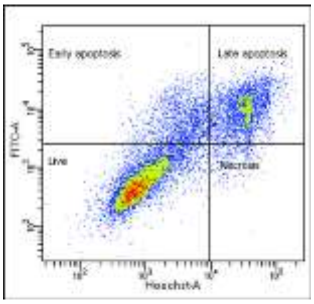	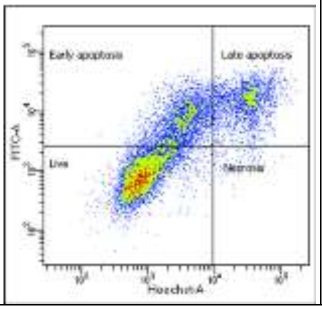	

Table S5. Effect of compounds **4a** and **5a** at concentrations 5 and 10 μg/ml on the distribution of HeLa cells in the cell cycle after 24h treatment.

Control	**4a**, 5 μg/ml	**5a**, 5 μg/ml
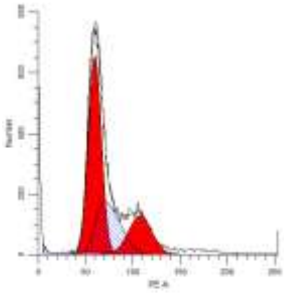	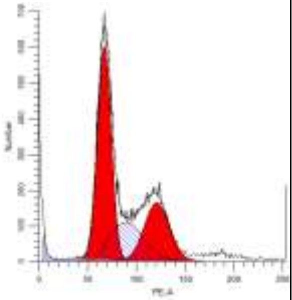	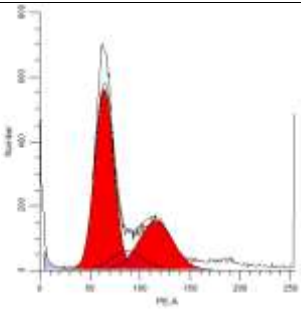
	**4a**, 10 μg/ml	**5a**, 10 μg/ml
	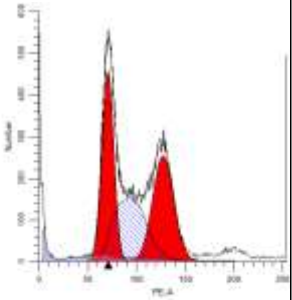	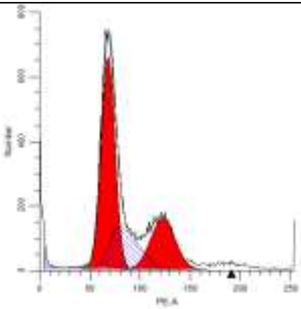

	Control	**4a** (5 μg/ml)	**4a** (10 μg/ml)	**5a** (5 μg/ml)	**5a** (10 μg/ml)
SubG1 (%)	2.41±0.19	4.22±0.25	6.07±0.83	1.08±0.12	4.21±0.51
G0/G1 (%)	53.03±1.67	49.75±1.33	31.82±3.18	59.91±1.45	51.86±2.13
S (%)	26.38±1.05	21.73±1.36	30.83±2.79	10.22±0.62	21.59±1.46
G2/M (%)	18.85±0.93	24.30±1.25	31.73±3.02	28.79±1.48	22.34±1.67

Table S6. Effect of compounds **4a** and **5a** at concentrations 5 and 10 μg/ml on the distribution of K562 cells in the cell cycle after 24h treatment.

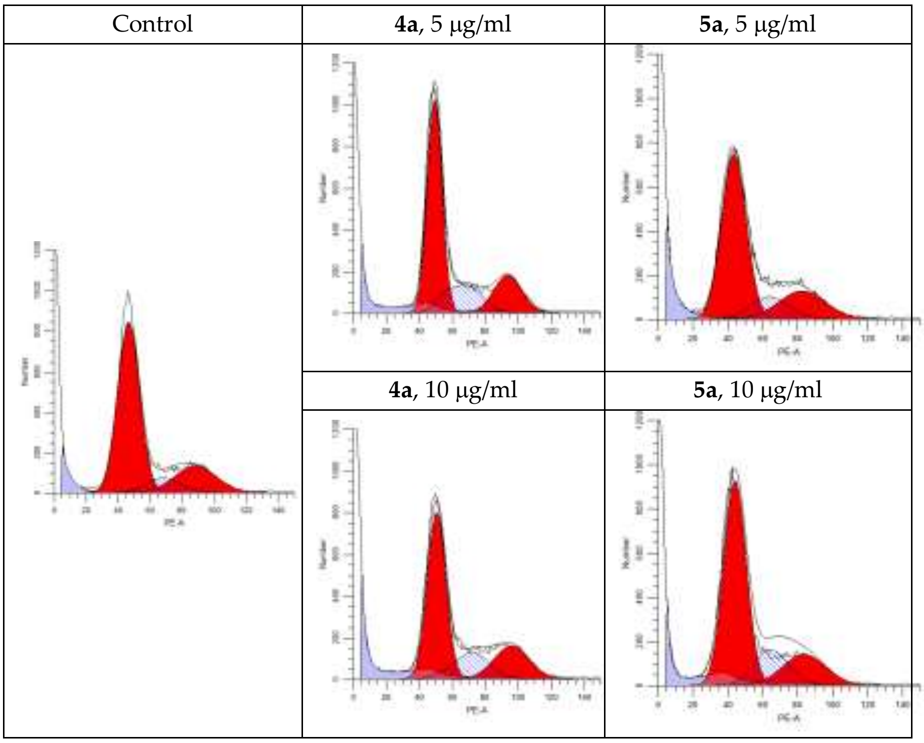

	Control	**4a** (5 μg/ml)	**4a** (10 μg/ml)	**5a** (5 μg/ml)	**5a** (10 μg/ml)
SubG1 (%)	3.62±0.23	11.32±0.95	14.36±1.22	9.82±0.70	10.31±1.21
G0/G1 (%)	67.51±1.12	53.04±1.01	50.62±1.92	58.91±1.72	57.78±2.04
S (%)	8.93±0.74	17.91±1.07	15.60±0.98	12.31±1.13	15.35±2.99
G2/M (%)	19.94±1.12	17.73±0.95	19.15±2.25	18.96±1.52	16.56±2.16

Table S7. IC_{50} values (μM) of screened compounds for K562 and HeLa cell line.

	**4a**	**5a**	**4b**	**5b**	**4c**	**5c**	**4d**	**5d**
**K562**	4.8±0.2	37.4±0.5	18.3±0.4	110.3±0.7	>100	>100	40.8±0.7	>100
**HeLa**	17.3±0.4	40.7±0.5	69.7±0.7	109.9±1.1	–	–	–	–

Table S8. The values of the signal intensities calculated by processing the images of the obtained Western blots in ImagJ.

	**4a**	**5a**	Control
GAPDH	14617<br>14342<br>14584	13427<br>13381<br>13042	12034<br>11721<br>12148
p53	12939<br>12847<br>13445	3826<br>3836<br>4218	3457<br>3244<br>3663
MDM2	8475<br>10399<br>7096	11781<br>12466<br>12518	16474<br>17393<br>16723
